# High prevalence of extended-spectrum beta-lactamase-producing Gram-negative pathogens from patients attending Felege Hiwot Comprehensive Specialized Hospital, Bahir Dar, Amhara region

**DOI:** 10.1371/journal.pone.0215177

**Published:** 2019-04-15

**Authors:** Feleke Moges, Setegn Eshetie, Wondwossen Abebe, Feleke Mekonnen, Mulat Dagnew, Abyot Endale, Azanaw Amare, Tigist Feleke, Mucheye Gizachew, Moges Tiruneh

**Affiliations:** 1 Department of Medical Microbiology, School of Biomedical and laboratory Sciences, College of Medicine and Health Sciences, University of Gondar, Gondar, Ethiopia; 2 Department of Pharmacognosy, School of Pharmacy, College of Medicine and Health Sciences, University of Gondar, Gondar, Ethiopia; 3 Department of Hospital Laboratory, University of Gondar Comprehensive Specialized Hospital, Gondar, Ethiopia; Ross University School of Veterinary Medicine, SAINT KITTS AND NEVIS

## Abstract

**Background:**

Infections caused by extended-spectrum beta-lactamases (ESBL) producing Gram-negative bacteria has emerge as a global threat in clinical practices. The treat is more serious in developing countries due to inappropriate use, poor adherence, use of counterfeit, sub-standard antibiotics and poor infection control practices. Data on ESBL producing Gram-negative bacteria are limited in developing countries including Ethiopia. The aim of this study was therefore, to describe the burden of ESBL producing Gram negative pathogens isolated from patients attending at Felege Hiwot Comprehensive Specialized Hospital, Bahir Dar, Amhara region.

**Materials and methods:**

A total of 532 clinical samples of blood, urine, stool, wound, abscess, ear discharge, nasal discharge, cervical discharge and body fluid specimens were aseptically collected and bacteriologically processed. Identification of the bacterial species was performed using an automated system (Vitek-2 Compact 27530, USA) and antibiotic susceptibility test was determined by disk diffusion method and selection of antibiotics were in accordance with CLSI guidelines. The MDR pattern of the Gram-negative pathogens was assessed using phenotypic methods of ESBL and carbapenemase production following standard procedure.

**Result:**

A total of 532 samples were processed and 263 pathogens were isolated. Of these, 185 (70.3%) were Gram-negative and 78 (29.7%) Gram-positive. Of the Gram-negative bacteria the high proportion of the isolates were identified from blood 146/185 (78.9%) and 29/185 (15.7%) were from urine cultures. The most common isolate in all clinical samples was *Klebsiella pneumoniae* 97/185 (52.4%) followed by *Escherichia coli 23*/185 (12.4%), *Acinetobacter baumannii 15*/185 (17.6%) and *Enterobacter aerogenes 12*/185(6.5%). Of the total Gram negatives, the prevalence of MDR was 148/185 (80.0%). Of the MDR isolates the prevalence of ESBL producers were, 127/148 (85.8%) and 24/148 (16.2%) were carbapenemase producers.

**Conclusion and recommendation:**

Prevalence of MDR and ESBL producing Gram-negative pathogens in this hospital is alarmingly high. Therefore, continuous monitoring of the problem with effective infection prevention and careful selection of empirical therapy are warranted in the study area.

## Introduction

Antibiotics play a critical role in reducing the burden of communicable diseases all over the world. Among the many classes of antibiotics, the β-lactam antibiotics are the most commonly used because of their broad-spectrum activities and better safety profiles [1]. However, emergence of antibiotic resistance threatens the effectiveness of successful treatment of infections and is a public health issue with national and global dimensions. The problem is more alarming in developing countries, where there is high burden of infectious diseases, lack of surveillance networks, laboratory capacity, and appropriate diagnostics [2,3]. Recent advances in drug resistance revealed that emergence of extended spectrum β-lactamase producing *Enterobacteriaceae* are increasing from time to time. Gram-negative bacilli like *Escherichia coli*, *Klebsiella pneumoniae*, *Pseudomonas aeruginosa*, and *Acinetobacter* spp. are important pathogens and may cause blood stream, abdominal and urinary tract infections. A few studies in Africa reported high case-fatality rate associated with blood stream infection, mainly caused by Gram-negative bacteria, and being 2-fold higher than that of malaria (43.5% versus 20.2%) [4, 5].

The overall pooled estimate of ESBL proportion in East African hospitals reported to be 42% [6]. A recent report from Addis Ababa, Ethiopia also confirm that there is a high prevalence of ESBL producing *Enterobacteriaceae* (78.7%) reported from blood stream and urinary tract infections [7]. However, there is little information about ESBL in developing countries like Ethiopia. Therefore, it is crucial to continuously monitor and evaluate the threat posed by ESBL in the community and aware the policymakers and health care professionals to prioritize the public health problems in the region. The aim of the present study was therefore, to evaluate the burden of ESBL at Felege Hiwot Comprehensive Specialized Hospital, Bahir Dar, Amhara region.

## Materials and methods

### Study area, design and period

The study was conducted at Felege Hiwot Comprehensive Specialized Hospital, Bahir Dar, Amhara region. Bahir Dar is a capital city of Amhara region, in the Northwest of Ethiopia. The city is located 565 kms far from Addis Ababa, capital city of Ethiopia. It has a total projected population of 649,426 (city health office 2018 report). Felege Hiwot Comprehensive Specialized Hospital is a tertiary referral hospital with around 423 beds and provide specialized and referral health care services for more than 7 million people who are living in the city and its surrounding regions. The study was an institution based cross-sectional study, conducted from December 2017 to April 2018.

## Ethical approval and consent to participate

Ethical approval was obtained from the institutional review board of the University of Gondar with reference number O/VIP/RCS/05/478/2015. Informed written consent was obtained from each study participants. Children less than 18 years who are not able to give consent were also asked an assent and/or written consent taken from their parents or guardians.

### Source and study populations

The source population was all patients who were attending the Felege Hiwot Comprehensive Specialized Hospital seeking treatment during the study period. The study populations were all patients suspected for having bloodstream, UTI, wound and others infections at the Felege Hiwot Comprehensive Specialized Hospital during the study period.

### Sample size determination

A total of 532 study subjects were recruited in this study who were suspected for bacterial infections. Using convenient sampling technique, blood, urine, stool, wound, abscess, ear discharge, nasal discharge, cervical discharge and body fluid specimens were aseptically collected from study participants for cultures, from December 2017 to April 2018.

### Sample processing and isolation of pathogens from clinical samples

#### Blood culture

Blood samples of 10 ml from adults, 5ml from pediatrics age group and 2ml from neonates were aseptically collected in duplicate (2 bottles for each patient at different time). The blood samples were inoculated in tryptic soya broth and incubated immediately aerobically at 35–37°C for 18hrs. After 18 hour of incubation, Gram stain was done and sub-cultured on to 5% Chocolate agar and MacConkey agar. The chocolate agar plates were incubated in a carbon dioxide atmosphere for up to 48 hours, and the MacConkey agar plate aerobically overnight. Those blood culture bottles which do not show growth, were continuously monitored for potential growth of pathogens until 7 days and if no growth after 7days, the blood culture were reported as negative [8].

#### Urine samples

Mid-stream urine specimens were collected using wide sterile mouth cupped and inoculated onto blood agar, Cysteine Lactose Electrolyte Deficient agar (CLED) and incubated overnight at 37°C using calibrated loop (measures 1.3mm diameter, delivering 1 μL). The samples with significant bacteriuria (= 10^5^ CFU/mL) were further processed.

**Other samples**

Bacteria from other clinical samples such as stool, wound and other body fluids were processed following standard procedures [8].

All isolates of Gram-negative bacteria were subjected and identified using an automated system (Vitek-2 Compact 27530, USA) and antibiotic susceptibility were performed by disk diffusion methods and selection of antibiotics were in accordance with CLSI guidelines.

### Antibiotic susceptibility testing

Antibiotic susceptibility testing was performed to all isolates by using the disk diffusion method against; ampicillin (AMP, 10μg), amoxicillin- clavulanic acid (AMC, 20/10μg), cotrimoxazole (SXT, 25μg), tetracycline (TET, 30μg), ciprofloxacin (CIP,5μg), chloramphenicol (CHL, 30μg), gentamycin (GEN, 10μg), cefepime (FEP, 30μg), cefixime (CFM 5μg), ceftriaxone (CRO, 30μg), cefoxitin (FOX, 30μg), and ceftazidime (CAZ, 30 μg) (all from Abtekbio.Ltd UK) were selected in accordance with CLSI guidelines. Bacterial inocula were prepared by suspending the freshly grown bacteria in 3–5 ml normal saline and turbidity was adjusted to 0.5 McFarland standards. Sterile cotton swab was dipped and rotated several times, and was pressed against the wall of the test tube. It was then swabbed over the entire surface of the agar. Antimicrobial impregnated paper disks were placed on the plate and incubated aerobically at 37°C for 24 hours. The zone of inhibition was measured by calibrated ruler and interpreted as sensitive, intermediate or resistant by using standard chart [9].

#### Screening for ESBL

This was done as part of the routine susceptibility testing, according to criteria recommended by the CLSI. The disk diffusion technique was used for screening ESBL using ceftazidime (30 μg) and cefotaxime (30 μg). If the result showed an inhibition zone of < 22 mm for ceftazidime and < 27 mm for cefotaxime indicated that the strain probably produces ESBL [9].

#### Phenotypic confirmatory test for ESBL production

This was done as per CLSI recommendations using double disk diffusion method, on Mueller–Hinton agar (OXOID. Ltd. England). Four discs, containing cefotaxime (30μg), cefotaxime/clavulanic acid (30 μg/ 10 μg), ceftazidime (30 μg) and ceftazidime/clavulanic acid (30 μg / 10 μg), were used (OXOID. Ltd. England). Inhibition zone ≥ 5 mm increase in either antimicrobial tested in combination with clavulanic acid versus its zone when tested alone confirmed ESBL production [9].

#### Modified Hodge test (MHT)

Carbapenemase producing isolates of Gram-negative bacteria were phenotypically investigated by MHT. A 0.5 McFarland suspension of carbapenem susceptible strains of *E*. *coli* ATCC25922 was used as a lawn over Mueller Hinton agar plates and meropenem (10 μg) disc were placed at the center of the plates. Isolates to be tested along with a positive control using *K*. *pneumoniae* ATCC1705. Inoculated plates were kept for 15 minutes at room temperature and incubated at 37°C for 24 hours. Observing a clover leaf like shape was considered as positive for carbapenemase production.

### Quality control

The reliability of the findings was guaranteed by implementing quality control measures throughout the whole processes of the laboratory work. The reference strains used as control were *E*. *coli* (ATCC 25922), *K*. *pneumoniae* (ATCC1705).

### Data analysis

Data were entered and analyzed using SPSS version 20. Simple frequency was applied to see the distribution of sociodemographic variables.

## Results

Of the 532 study participants enrolled in the present study, 290 (54.5%) were male, 263 (49.4%) were children aged less than 6 years and only 8 (1.5%) were greater than 60 years. Three hundred and nine (58.9%) were rural dwellers and 459 (86.3%) were inpatients. As most of the study participants came from rural areas, 99 (18.6%) of the study participants were illiterate. Regarding to patient setting majority of the patients were admitted patients, 459 (86.3%). (Table 1).

**Table 1:** Sociodemographic characteristics of patients attending at Felege Hiwot Comprehensive Specialized Hospital, Amhara region, 2017–18.

Socio-demographic characteristics		Frequency	Percentage (%)
**Sex**	**Male**	**290**	**54.5**
	**Female**	**242**	**45.5**
**Age**	**≤ 5**	**263**	**49.4**
	**6–15**	**69**	**13.0**
	**16–30**	**136**	**25.6**
	**31–45**	**39**	**7.3**
	**46–60**	**17**	**3.2**
	**>60**	**8**	**1.5**
**Residence**	**Urban**	**223**	**41.9**
	**Rural**	**309**	**58.9**
**Educational status**	**Illiterate**	**99**	**18.6**
	**Primary**	**54**	**10.2**
	**Secondary**	**58**	**10.9**
	**Diploma and above**	**58**	**10.9**
	Not applicable*	**263**	**49.4**
**Patient setting**	**OPDs**	**73**	**13.7**
	**Wards**	**459**	**86.3**
**Total**		**532**	**100**

* Children ≤ 5 years

Of the total of 532 samples processed for culture, 388 (72.9%) blood, 81 (15.2%) urine and 63 (11.8%) were other samples. Of these 532 samples, 263 (49.4%) were culture positive. Of the total isolates, 185 (70.3%) were Gram-negative and 78 (29.7%) were Gram-positive (Table 2). The Gram-negative bacterial profiles revealed that, 146/185 (78.9%) were isolated from the blood samples, 29/185 (15.7%) were from the urine samples and 10/185 (5.4%) from other clinical samples (Table 3).

**Table 2:** Culture positivity rate of clinical samples collected at Felege Hiwot Comprehensive Specialized Hospital, Amhara region, 2017–18.

Clinical samples	Total sample cultured n = 532 (%)		
	Culture Negative n = 269 (%)	Culture Positive n = 263(%)	
		Gram Positives n = 78 (%)	Gram Negatives n = 185 (%)
**Blood (n = 388)**	**181 (34.0)**	**61 (23.2)**	**146 (55.5)**
**Urine (n = 81)**	**47 (8.8)**	**5 (1.9)**	**29 (11.0)**
**Stool (n = 32)**	**32 (6.0)**	**-**	**-**
**Wound (n = 10)**	**-**	**6 (2.2)**	**4 (1.5)**
**Abscess (n = 7)**	**1 (0.2)**	**4 (1.5)**	**2 (0.8)**
**Ear discharge (n = 5)**	**-**	**2 (0.8)**	**3 (1.2)**
**Body fluid (n = 1)**	**1(0.2)**	**-**	**-**
**Nasal discharge (n = 4)**	**3 (0.6)**	**-**	**1 (0.2)**
**Cervical discharge (n = 4)**	**4 (0.8)**	**-**	**-**
**Total (n = 532)**	**269 (50.6)**	**78 (29.7)**	**185 (70.3)**

**Table 3:** Bacterial profile of Gram-negative bacterial isolates at Felege Hiwot Comprehensive Specialized Hospital, Amhara region, 2017–18.

Clinical samples		Bacterial profile	Frequency	Percentage
Blood culture (n = 146)		*K*. *pneumoniae*	88	*47*.*7*
		*A*. *baumanii*	13	*7*.*0*
		*E*. *coli*	12	*6*.*7*
		*E*. *aerogenes*	7	3.8
		*Citrobacter spp*	7	*3*.*8*
		*E*. *cloacae*	7	*3*.*8*
		*P*. *aeruginosa*	5	*2*.*7*
		*K*. *oxytoca*	2	*1*.*1*
		*Salmonella spp*	2	*1*.*1*
		*Providencia stuartii *	1	0.5
		*Serratia* sp	1	0.5
		*Proteus sp*.	1	0.5
Urine culture (n = 29)		*E*. *coli*	10	5.4
		*K*. *pneumoniae*	7	3.8
		*E*. *aerogenes*	4	2.2
		*Citrobacter*	2	1.1
		*E*.* cloacae*	2	1.1
		*A*. *baumanii*	1	0.5
		*Salmonella sp*.	1	0.5
		*Providencia stuartii*	1	0.5
		*Proteus sp*.	1	0.5
Others (n = 10)	Wound	*K*. *pneumoniae*	1	0.5
		*Serratia sp*	1	0.5
		*A*. *baumanii*	1	0.5
		*Citrobacter* sp.	1	0.5
	Ear and nasal discharges	*K*. *pneumoniae*	1	0.5
		*Proteus* sp.	1	0.5
		*Citrobacter* sp.	1	0.5
		*P*. *aeruginosa*	1	0.5
	Abscesses	*E*. *coli*	1	0.5
		*E*. *aerogenes*	1	0.5
Total			185	100

The most common isolates in all clinical samples in the present study were *K*. *pneumoniae* 97/185 (52.4%) followed by *E*. *coli 23*/185 (12.4%), *A*. *baumanii 15*/185 (17.6%) and *E*. *aerogenes 12*/185(6.5%). Similarly, the most common isolates identified from the blood culture were *K*. *pneumoniae* 88/146 (60.3%) followed by *A*. *baumanii 13*/146 (8.9%), *E*. *coli* 12/146 (8.2%) and *E*. *aerogenes* 7/146 (4.8%) while *E*. *coli* 10/29 (34.5%) and *K*. *pneumoniae* 7/29 (24.1%) were common in the urine culture (Table 3).

One hundred forty-eight MDR isolates were identified, preserved and rechecked for different antibiotics (12 antibiotics), screened for extended spectrum beta-lactamase production and Modified Hoge test for carbapenemase production. Among these isolates, 127(85.8%) were ESBL producers and 24 (16.2%) were carbapenemase producers (Table 4). One hundred and five (70.9%) of the isolates from blood samples and 20 (13.5%) from urine samples were ESBL producers. Of the blood culture isolates, 22/148 (14.9%) were carbapenemase producers and 2/148 (1.4%) were from urine cultures (Table 4). Of the 148 isolates screened for ESBL, 79 (53.3%) were *K*. *pneumoniae* followed by 14 (9.4%) *E*. *coli* and 9 (6.0%) *E*. *aerogenes*. Comparing with other isolates, relatively high number of *K*. *pneumoniae* 16 (10.8%) were carbapenemase producers than *Enterobacter cloacae* 3 (2.0%) and *E*. *coli* 2 (1.4%) (Table 4).

**Table 4:** Bacterial profile, ESBL and carbapenamse production of Gram-negative bacteria in relation to clinical samples collected at Felege Hiwot Comprehensive Specialized Hospital, Amhara region, 2017–18.

Organism	Clinical sample	ESBL Production (n = 148)		Carbapenmase production (n = 148)		Total
		Positive (%)	Negative (%)	Positive (%)	Negative (%)	
*K*. *pneumoniae*	Blood	72 (48.6)	5 (3.4)	15 (10.1)	62 (41.9)	77 (52.0)
	Urine	6 (4.1)	-	1 (0.7)	5 (3.4)	6 (4.1)
	Wound	-	1(0.7)	-	1 (0.7)	1 (0.7)
	Ear discharge	1(0.7)	-	-	1 (0.7)	1 (0.7)
	Total	79 (53.3)	6 (4.1)	16 (10.8)	69 (46.6)	85 (57.4)
*E*. *coli*	Blood	8 (5.4)	4 (2.7)	1 (0.7)	11 (7.4)	12 (8.1)
	Urine	5 (3.4)	1 (0.7)	1 (0.7)	5 (3.4)	6 (4.1)
	Abscess	1 (0.7)	-	-	1 (0.7)	1 (0.7)
	Total	14 (9.5)	5 (3.4)	2 (1.4)	17 (11.5)	19 (12.8)
*E*. *aerogenes*	Blood	5 (3.4)	1 (0.7)	-	6 (4.1)	6 (4.1)
	Urine	4 (2.7)	-	-	4 (2.7)	4 (2.7)
	Total	9 (6.0)	1 (0.7)	-	10 (6.7)	10 (6.7)
*A*. *boumannii*	Blood	6 (4.1)	1 (0.7)	1 (0.7)	6 (4.1)	7 (4.7)
	Urine	-	1 (0.7)	-	1 (0.7)	1 (0.7)
	Total	6 (4.1)	2 (1.4)	1 (0.7)	7 (4.7)	8 (5.4)
*Citrobacter* spp.	Blood	5 (3.4)	-	1 (0.7)	4 (2.7)	5 (3.4)
	Urine	2 (1.4)	-	-	2 (1.4)	2 (1.4)
	Total	7 (4.7)	-	1 (0.7)	6 (4.1)	7 (4.7)
*E*. *cloacae*	Blood	4 (2.7)	1 (0.7)	3 (2.0)	2 (1.4)	5 (3.4)
	Urine	2 (1.4)	-	-	2 (1.4)	2 (1.4)
	Total	6 (4.1)	1 (0.7)	3 (2.0)	4 (2.7)	7 (4.7)
*Salmonella* spp.	Blood	1 (0.7)	1 (0.7)	1 (0.7)	1 (0.7)	2 (1.4)
	Urine	-	1 (0.7)	-	1 (0.7)	1 (0.7)
	Total	1 (0.7)	2 (1.4)	1 (0.7)	2 (1.4)	3 (2.0)
*P*. *aeruginosa*	Blood	2 (1.4)	1 (0.7)	-	3 (2.0)	3 (2.0)
*K*. *oxytoca*	Blood	2 (1.4)	-	-	2 (1.4)	2 (1.4)
*Providencia stuartii*	Blood	-	1 (0.7)	-	1 (0.7)	1 (0.7)
	Urine	1 (0.7)	-	-	1 (0.7)	1 (0.7)
	Total	1 (0.7)	1 (0.7)		2 (1.4)	2 (1.4)
*Serratia* spp	Blood	-	1 (0.7)	-	1 (0.7)	1 (0.7)
	Wound	-	1 (0.7)	-	1 (0.7)	1 (0.7)
	Total		2 (1.4)	-	2 (1.4)	2 (1.4)
Total		127 (85.8)	21 (14.2)	24 (16.2)	124 (83.8)	148 (100)

Among 148 Gram negative bacteria tested for multiple classes of antibiotics, all Gram-negative bacteria were resistant to ampicillin 148/148 (100%), followed by ceftazidime 143/148 (96.6%), cotrimoxazole 138/148 (93.2%), cefixime 137/148 (92.6) and cefepime 135/148 (91.2%). The least resistant rate was observed in cefoxitin 38/148 (25.7%) and ciprofloxacin 52/148 (35.1%). Cefoxitin was found to be better option of the cephalosporin antibiotics such as ceftazidime, cefixime, cefepime and ceftriaxone. All isolates of *E*. *aerogenes* and *E*. *cloacae* were resistant to cotrimoxazole and cephalosporin groups such as cefixime, cefepime, ceftriaxone, and ceftazidime (Table 5).

**Table 5:** Drug resistant patterns of Gram-negative bacterial isolates against commonly used antibiotic at Felege Hiwot Comprehensive Specialized Hospital, Amhara region, 2017–18.

Bacterial Isolates		AMP	AMC	SXT	TET	*CIP*	CHL	GEN	FEP	CFM	FOX	CRO	CAZ
*K*. *pneumoniae n = 85*	S	-	8 (9.4)	3 (3.5)	8 (9.4)	50 (58.8)	26 (30.6)	14 (16.5)	2 (2.4)	4 (4.7)	49 (57.6)	8 (9.4)	2 (2.4)
	I		14 (16.5)	-	-	13 (15.3)	5 (5.9)	2 (2.4)	3(3.5)	1 (1.2)	19 (22.4)	2 (2.4)	-
	R		63 (74.1)	82 (96.5)	77 (90.6)	22 (25.9)	54 (63.5)	69 (81.2)	80 (94.1)	80 (94.1)	17 (20.0)	75 (88.2)	83 (97.6)
*E*. *coli n = 19*	S		4 (21.1)	-	2 (10.5)	7 (36.8)	8 (42.1)	10 (52.6)	4 (21.1)	3 (15.3)	10 (52.6)	3 (15.3)	1 (5.3)
	I		3 (15.8)	-	-	-	3 (15.8)	-	-	1 (5.3)	4 (21.1)	-	1 (5.3)
	R	19 (100)	12 (63.2)	19 (100)	17 (89.5)	12 (63.2)	8 (42.1)	9 (47.4)	15 (79.0)	15 (79.0)	5 (26.3)	16 (84.2)	17 (89.5)
*E*. *aerogenes n = 10*	S	-	-	-	1 (10.0)	8 (80.0)	3 (30.0)	1 (10.0)	-	-	5 (50.0)	-	-
	I	-	-	-	-	1 (10.0)	2 (20.0)	1(10.0)	-	-	4 (40.0)	-	-
	R	10 (100)	10 (100)	10 (100)	9 (90.0)	1 (10.0)	5 (50.0)	8 (80.0)	10 (100)	10 (100)	1 (10.0)	10 (100)	10 (100)
*A*. *baumanii n = 8*	S	-	-	4 (50.0)	6 (75.0)	3 (37.5)	3 (37.5)	3 (37.5)	-	—	-	-	-
	I	-	-	-	-	2 (25.0)	-	-	2 (25.0)	-	2 (25.0)	2 (25.0)	-
	R	8 (100)	8 (100)	4 (50.0)	2 (25.0)	3 (37.5)	5 (62.5%)	5 (62.5%)	6 (75.0)	8 (100)	6 (75.0)	6 (75.0)	8 (100)
*Citrobacter s*pp *n = 7*	S	-	-	1 (14.3)	-	3 (42.9)	2 (28.6)	-	-	1 (14.3)	4 (57.1)	-	-
	I	-	-		-	1 (14.3)	-	-	1 (14.3)	-	2 (28.6)	1 (14.3)	-
	R	7 (100)	7 (100)	6 (85.7)	7 (100)	3 (42.9)	5 (71.4)	7 (100)	6 (85.7)	6 (85.7)	1 (14.3)	6 (85.7)	7 (100)
*E*. *cloacae n = 7*	S	-	-	-	-	1 (14.3)	-	1 (14.3)	-	-	1 (14.3)	-	-
	I	-	-	-	-	-	-	-	-	-	2 (28.6)	-	-
	R	7 (100)	7 (100)	7(100)	7 (100)	6 (85.7)	7 (100)	6 (85.7)	7 (100)	7 (100)	4 (57.1)	7 (100)	7 (100)
*P*. *aeruginosa n = 3*	S	-	-	-	1 (33.3)	1 (33.3)	1 (33.3)	-	-	-	-	-	-
	I	-	-	1 (33.3)	-	-	-	-	-	-	1 (33.3)	-	-
	R	3 (100)	3(100)	2 (66.7)	2 (66.7)	2(66.7)	2 (66.7)	3 (100)	3 (100)	3 (100)	2 (66.7)	3 (100)	3 (100)
*Salmonella spp*. *n = 3*	S	-	1 (33.3)	1 (33.3)	2 (66.7)	3 (100)	-	-	1 (33.3)	1 (33.3)	2 (66.7)	1 (33.3)	1 (33.3)
	I	-	-	-	-	-	-	-	-	-	1 (33.3)	-	-
	R	3 (100)	2 (66.7)	2 (66.7)	1 (33.3)		3 (100)	3 (100)	2 (66.7)	2 (66.7)		2 (66.7)	2 (66.7)
Others* *n = 6*	S	-	-	-	1 (16.7)	1 (16.7)	2 (33.3)	-	-	-	2 (33.3)	2 (33.3)	-
	I	-	1 (16.7)	-	-	2 (33.3)	-	-	-	-	2 (33.3)	-	-
	R	6 (100)	5 (83.3)	6(100)	5 (83.3)	3 (50.0)	4 (66.7)	6(100)	6 (100)	6 (100)	2 (33.3)	4 (66.7)	6(100)
*Total n = 148*	S		13 (8.8)	9 (6.1)	21 (14.2)	77 (52.0)	45 (30.4)	29 (19.6)	7 (4.7)	9 (6.1)	73 (49.3)	14 (9.5)	4 (2.7)
	I	-	18 (12.2)	1 (0.7)	-	19 (12.8)	10 (6.8)	3 (2.0)	6 (4.1)	2 (1.3)	37 (25.0)	5 (3.4)	1 (0.7)
	R	148 (100)	117 (79.0)	138 (93.2)	127 (85.8)	52 (35.1)	93 (62.8)	116 (78.4)	135 (91.2)	137 (92.6)	38 (25.7)	129 (87.2)	143 (96.6)

**Amp =** Ampicillin; **AMC = **Amoxicillin-clavulanic acid; **SXT =** Cotrimoxazole; **TET =** Tetracycline; **CIP =** Ciprofloxacin; **CHL =** Chloramphenicol; **GEN =** Gentamycin; **FEP =** Cefepime; **CFM =** Cefixime; **FOX =** Cefoxitin; CRO **=** Ceftriaxone; **CAZ =** Ceftazidime.

* *K*. *oxytoca (n = 2)*, *Serratia* spp (n = 2) and *Providencia stuartii* (n = 2).

Ciprofloxacin and cefoxitin were among the antibiotics which revealed a better sensitivity for *K*. *pneumoniae*. However, there is also high number of isolates with intermediate level of susceptibility observed in cefoxitin, 19/85 (22.4%) and ciprofloxacin, 13/85 (15.3%) compared with other antibiotics. Whereas, high level of intermediate susceptibility for isolates of *E*. *coli* was observed in cefoxitin, 4/19 (21.1%) and amoxicillin-clavulanic acid, 3/19 (15.8%). The overall rate of antibiotics that showed intermediate susceptibility were cefoxitin, 37/148 (25%) followed by ciprofloxacin, 19/148 (12.8%) and amoxicillin-clavulanic acid, 18/148 (12.2%) (Table 5)

Majority of the isolates were resistant to more than 9 antibiotics tested (Table 6). This pattern was also true for ESBL producing isolates. Of the total isolates, the rate of MDR were 148/185 (80.0%). The most common MDR isolates were *Klebsiella pneumoniae*,85/97(87.6%) followed by *E*. *aerogenes*, 10/12 (83.3%), *E*. *coli*, 19/23 (82.6%%), *E*. *cloacae*, 7/9 (77.8%) (Table 3).

**Table 6:** Multidrug resistant profile of Gram-negative bacteria isolated from clinical samples at Felege Hiwot Comprehensive Specialized Hospital, Amhara region, 2017–18.

Antibiogram pattern	Bacterial isolates*											Total N = 148
	*K*. *pneumoniae*	*E*. *coli*	*E*. *aerogenes*	*A*. *baumanii*	*E*. *cloacae*	*Citrobacter spp*.	*Salmonella spp*	*P*. *aeruginosa*	*K*. *oxytoca*	*Providencia stuartii *	*Serratia* spp	
AMC, SXT, TET		1										1
SXT, TET, CHL, GEN	1						1					2
SXT, TET, CHL, CFM, CAZ		3										3
SXT, CIP, GEN, FEP, CFM, CRO, CAZ	1											1
SXT, TET, GEN, FEP, CFM, FOX, CAZ	2											2
AMC, TET, FEP, CFM, CRO, FOX, CAZ	1		1	2								4
AMC, SXT, CHL, FEP, CFM, CRO, CAZ	1	1										2
AMC, SXT, TET, FEP, CFM, CRO, CAZ	3										1	4
AMC, SXT, TET, CAF, FEP, CRO, CAZ	2											2
SXT, TET, CHL, GEN, FEP, CFM, CRO, CAZ	4											4
AMC, SXT, GEN, FEP, CFM, CRO, FOX, CAZ	2						1					3
AMC, SXT, CHL, GEN, FEP, CFM, CRO, CAZ	1	2					1					4
AMC, SXT, TET, FEP, CFM, CRO, FOX, CAZ	1		2									3
AMC, SXT, TET, GEN, FEP, CFM, CRO, CAZ	3					1					1	5
AMC, SXT, TET, GEN, FEP, CFM, CRO, FOX, CAZ	5	1										6
AMC, SXT, TET, CHL, FEP, CFM, CRO, FOX, CAZ	1		3			1			1			6
AMC, SXT, TET, CHL, GEN, FEP, CFM, CRO, CAZ	18	1								1		20
AMC, SXT, TET, CIP, GEN, FEP, CFM, CRO, CAZ	8	1										9
AMC, SXT, TET, CIP, FEP, CFM, CRO, FOX, CAZ		2		1								3
AMC, SXT, TET, CHL, GEN, FEP, CFM, CRO, FOX, CAZ	7	1	2	1	1	1		2				15
AMC, SXT, TET, CIP, GEN, FEP, CFM, CRO, FOX, CAZ	2	2		4		1				1		10
AMC, SXT, TET, CIP, CHL, FEP, CFM, CRO, FOX, CAZ	1	1			1							3
AMC, SXT, TET, CIP, CHL, GEN, FEP, CFM, CRO, CAZ	6	1			1	2						10
AMC, SXT, TET, CIP, CHL, GEN, FEP, CFM, CRO, FOX, CAZ	15	2	2		4	1		1	1			26
Total	85	19	10	8	7	7	3	3	2	2	2	148

**AMC =** Amoxicillin-clavulanic acid; **SXT =** Cotrimoxazole; **TET =** Tetracycline; **CIP =** Ciprofloxacin; **CHL =** Chloramphenicol; **GEN =** Gentamycin; **FEP =** Cefepime; **CFM =** Cefixime; **FOX =** Cefoxitin; **CRO =** Ceftriaxone; **CAZ =** Ceftazidime.

*In vitro drug susceptibility testing was not performed for intrinsically resistant isolates.

## Discussions

Half of the clinical samples processed in our study were culture positive of which the Gram- negative pathogens were in the ratio of 3.3:1 compared with the Gram-positive isolates. This is similar to a study done in Iran that the ratio of Gram-negative to Gram-positive bacteria was 3.2:1 [10]. As pointed out by Abe *et*.*al* [11], the reason may be because Gram-negative bacteremia induces greater magnitude of inflammatory response than Gram-positive bacteremia as a result C-reactive protein and IL-6 levels are significantly higher in Gram-negative bacteremia than in Gram-positive bacteremia. These observations suggest a distinct immuno-pathophysiologic behavior of sepsis in patients with Gram-negative bacteremia that may influence clinical outcomes.

*K*. *pneumoniae*, *E*. *coli*, *A*. *baumanii* and *E*. *aerogenes* were the most common Gram-negative isolates in all clinical samples of the present study. Similarly, a study on septicemia revealed that *E*. *coli* (27/70, 38.6%), *K*. *pneumoniae* (24/70, 34.3%), *A*. *baumanii* (9/70, 12.8%) were the commonest Gram- negative isolates [12].

The most common isolates from blood culture were *K*. *pneumoniae* 77 (52.0%) followed by *E*. *coli* 12 (8.1), *A*. *baumanii*, 7 (4.7) and *E*. *aerogenes*, 6(4.0). Similar study from Iran revealed that, *Klebsiella pneumoniae* 52 (33.5%), *E*. *coli*, 32 (20.6%) and *Enterobacter* sp., 15 (9.7%) in blood cultures [13] and in Pakistan, *Klebsiella*, *E*. *coli*, *Enterobacter* remains the principal Gram-negative bacteria responsible in blood stream infections [14].

Of the isolates screened for ESBL, 79 (53.3%) *K*. *pneumoniae*, 14 (9.4%) *E*. *coli* and 9 (6.0%) *E*. *aerogenes* were predominant. A report from Brazil showed that, similar patterns of ESBL producing *K*. *pneumoniae* (53.8%) and high rate *E*. *coli* (100%), *E*. *aerogenes (100%)* ESBL producers [15]. In addition, different studies in Kuwait confirm that, *K*. *pneumoniae* (82.1%) and *E*. *coli* (62%) [16], Albania 85.7% of *E*. *coli* and 100% *K*. *pneumoniae* [17] and Tanzania, *K*. *pneumoniae* 11 (69%) and *E*. *coli* 12 (92.3%) isolates were ESBL producers [18]. The difference in these results may be due to geographical variation, the diagnostic methods and the difference in antibiotic practices of the study area.

The overall prevalence of ESBL production in the present study was 127 (85.8). This was higher than study done in Tanzania where the rate of ESBL producing Enterobacteriaceae were 79.3% [18] and Addis Ababa, 78.6% [7].

The overall prevalence carbapenemase production in the present study was 24 (16.2). The carbapenemase producing isolates reported in different hospital settings ranged from 2.3% to 67.7% in North Africa and from 9% to 60% in sub-Saharan Africa [19]. It was lower than the studies from China carbapenem-resistant *Klebsiella pneumoniae* (CRKp) to be 31 (65%) [20] and slightly higher in Addis Ababa, 12.1% [7].

Comparing with other isolates relatively high number of isolates of *K*. *pneumoniae* 16 (10.8%) were carbapenamse producers. This is lower than a study from Brazil where 50% of *K*. *pneumoniae* isolates were positive for carbapenemase production [15].

Among 148 Gram negative bacteria tested for multiple classes of antibiotics all isolates of Gram-negative bacteria were resistant to ampicillin 147/148 (100%), followed by ceftazidime 143/148 (96.6%), cotrimoxazole 138/148 (93.2%), cefixime 137/148 (92.6), cefepime 135/148 (91.2%) and least resistant to cefoxitin 38/148 (25.7%) and ciprofloxacin 52/148 (35.1%). Similar finding was reported in Saudi Arabia for isolates of *E*. *coli* to some of the antibiotics we reported also high resistant to ampicillin (96.61%), ciprofloxacin (79.66%), cefepime (75.58%), aztreonam (89.83%), cefotaxime (76.27%), ceftazidime (81.36%) and less resistant to cefoxitin (15.25%) [21]. The high resistant variation observed to ciprofloxacin may be due to its affordability and availability variation in the two study areas.

Cefoxitin was showing better performance than other members of cephalosporin such as ceftazidime 143/148 (96.6%), cefixime 137/148 (92.6), cefepime 135/148 (91.2%) and ceftriaxone 129/148 (87.2%). High rate of resistant to cephalosporin group was also reported from isolates of *K*. *pneumoniae* in India (>90%) [22] and as reported by Tohamy *et al* from Egypt, all MDR Gram-negative showed high resistant to ampicillin, cefepime ceftriaxone, and cephradine [12]. Cefoxitin was found to be better option of the cephalosporin antibiotics, which was also supported by other study [21].

*K*. *pneumoniae*, *E*. *aerogenes*, *E*. *coli*, and *E*. *cloacae* were the commonest MDR isolates in our study. Majority of the isolates were resistant to more than 9 antibiotics tested. This pattern was also true for ESBL producing isolates and the overall MDR rate was 80.0%. This was similar to a study done at Mekele Hospital, where 82.9% multi drug resistant to the commonly used antibiotics [23]. However, it was higher than a study in Marikos, where 72.2% [24] and Tanzania, 61.4%, [18] but lower than a study from Jimma, 85% [25]. Many of the isolates in the present study becomes resistant to commonly used antibiotics. This high prevalence of MDR in the present study may be due to the reason that, majority of our study subjects were admitted patients (83.6%) where many antibiotics are circulating in the hospital which may serve as a selective pressure for increasing MDR isolates.

## Conclusion and recommendations

The overall MDR isolates in the present study was 80%. Among phenotypically tested Gram-negative bacteria 85.8% were ESBL producers and 16.2% were carbapenemase producers. The Gram negatives showed high level of drug resistance towards the tested cephalosporin except cefoxitin. Although there is an increasing intermediate resistance in cefoxitin and ciprofloxacin, compared to other antibiotics tested; these antibiotics holds promise as an alternative choice of therapy for Gram-negative bacterial infections. Finding high rate of ESBL production in Gram-negative bacteria require strict infection control measures and careful selection of empirical therapy in the study area.

## Supporting information

S1 Questionnaire Survey of antibacterial drug resistance in Amhara regional state referral hospitals, Bahir Dar, Ethiopia. (DOCX)

S1 Data Raw data. (PDF)

## Abbreviations

ESBL: Extended spectrum β-Lactamase
MDR: Multi-drug resistant
UTI: Urinary tract infection
