# Supplementary material for: High prevalence of extended-spectrum beta-lactamase-producing Gram-negative pathogens from patients attending Felege Hiwot Comprehensive Specialized Hospital, Bahir Dar, Amhara region
Source: PLoS One. 2019 Apr 15;14(4):e0215177. doi: 10.1371/journal.pone.0215177 (PMC6464180; doi:10.1371/journal.pone.0215177)
Supplement: S1 Questionnaire — (DOCX) [file pone.0215177.s001.docx]

**Questionnaire**

***Survey of antibacterial drug resistance in Amhara regional state referral hospitals, Bahir Dar, Ethiopia***

| Participant’s ID No. _____________________Hospital No._____________________Date___________________ | |
| --- | --- |
| **Questions** | **Response categories** |
| 1. Sex | Male …………….1 Female ………….2 |
| 1. Age _______years |  |
| 1. Are you on antibacterial treatment? | Yes………………1 No………………..2 |
| 1. If yes for Q#3, for how long? | .______________days |
| 1. Current residence: | Urban ……………1 Rural……………..2 |
| 1. Have you ever attended school? | Yes…………….1 No ……….……2 |
| 1. What is the highest level of school you attended? | Primary ……….1 Secondary …....2  Higher………..3 |
| 1. What is your current occupation? | Employed ……….1 Merchant………...2  House wife ...........3 Daily laborer …….4  Farmer……………5 Other (specify)..........................6 |
| 1. Have you ever been sick and had visited a health professional? | Yes ……………..1 No………….......2 |
| 1. If yes for Q#9, list the diseases |  |
| 1. Have you ever taken an antimicrobial treatment? | Yes …………….1 No………………2 |
| 1. If yes for Q#11, list the antimicrobials |  |
| 1. Did you ever stop taking an antimicrobial drugs before you completed? | Yes……………..1 No……………..2 |
| 1. Why did you stop taking the antimicrobial by yourself? | Didn’t have enough money………..1  Was told to stop by health worker…2  Condition didn’t improved…………3  Condition improved …………….….4  Other specify ……………………….5 |
| 1. Some antimicrobial drugs that used to work in fighting infections no longer work. This problem is called antimicrobial resistance. Have you heard of this problem before? | Yes ……………..1 No………..…..….2 |
| 1. If yes for Q#15, what can cause antimicrobial drugs to stop working? | Poor quality of antimicrobials ………1  Insufficient amount of antimicrobials...2  When someone uses the wrong antimicrobials ……………………..3  When someone stop taking it before supposed to do so…………………….4  When someone use antimicrobials without prescription ………………….5  When someone uses antimicrobials that are prescribed for someone else………6  Other (specify) ……………………….7 |
| Current patient information |  |
| 1. Patient setting | OPD …………………….1 Ward …………………….2 |
| 1. Reason for hospital admission | AFI……………………….1  Pneumonia ……………….2  UTI………………………..3  Meningitis…………………4  Gastroenteritis……………...5  Other specify ………….……6 |
| 1. Ward in which the patient is admitted to | Pediatrics ………………...1  Gny Obs……………..…...2  Surgical………………..…3  Medical ……………….…4  ICU ……………………....5  ANC …………………..…6  Emergency……………..…7  Neonatology………………8 |
| 1. For how long have you been admitted for this case? | ________day/s |
| 1. Type of Clinical sample (s) | _____________________________________________ |
