# Supplementary material for: High prevalence of extended-spectrum beta-lactamase-producing Gram-negative pathogens from patients attending Felege Hiwot Comprehensive Specialized Hospital, Bahir Dar, Amhara region
Source: PLoS One. 2019 Apr 15;14(4):e0215177. doi: 10.1371/journal.pone.0215177 (PMC6464180; doi:10.1371/journal.pone.0215177)
Supplement: S1 Data — (PDF) [file pone.0215177.s002.pdf]

ID	MRN	Sex	Age	residence	School	level of school	occupation	patient setting	ward
1.00	#######	2.00	3.00	2.00	.00	.00	5.00	2.00	1.00
4.00	#######	2.00	1.00	2.00	.00	.00	5.00	2.00	1.00
5.00	1101.00	1.00	1.00	1.00	.00	.00	1.00	2.00	1.00
6.00	1102.00	1.00	1.00	2.00	.00	.00	-1.00	2.00	5.00
7.00	1094.00	1.00	1.00	2.00	.00	.00	5.00	2.00	5.00
8.00	1103.00	2.00	3.00	2.00	.00	.00	5.00	2.00	5.00
9.00	1104.00	2.00	1.00	2.00	.00	.00	5.00	2.00	5.00
10.00	1105.00	2.00	1.00	2.00	.00	.00	5.00	2.00	5.00
11.00	1117.00	1.00	1.00	2.00	.00	.00	-1.00	2.00	1.00
12.00	1119.00	1.00	1.00	1.00	.00	.00	-1.00	2.00	5.00
2.00	#######	1.00	1.00	2.00	.00	.00	5.00	2.00	1.00
3.00	#######	1.00	1.00	2.00	.00	.00	5.00	2.00	1.00
13.00	1116.00	1.00	1.00	2.00	.00	.00	5.00	2.00	1.00
14.00	1122.00	2.00	1.00	2.00	.00	.00	5.00	2.00	5.00
15.00	1127.00	1.00	2.00	2.00	.00	.00	5.00	2.00	1.00
16.00	1120.00	2.00	13.00	1.00	1.00	1.00	6.00	2.00	1.00
17.00	1121.00	1.00	1.00	1.00	.00	.00	2.00	2.00	1.00
18.00	1124.00	2.00	1.00	1.00	.00	.00	-1.00	2.00	5.00
19.00	1130.00	2.00	21.00	1.00	.00	.00	5.00	2.00	1.00
20.00	1131.00	2.00	40.00	2.00	.00	.00	5.00	2.00	4.00
21.00	1133.00	2.00	2.00	2.00	.00	.00	5.00	2.00	1.00
22.00	1139.00	1.00	1.00	1.00	.00	.00	1.00	2.00	5.00
23.00	1140.00	1.00	14.00	2.00	.00	.00	5.00	2.00	4.00
24.00	1134.00	1.00	1.00	2.00	.00	.00	5.00	2.00	5.00
25.00	1150.00	1.00	1.00	2.00	.00	.00	5.00	2.00	5.00
26.00	1156.00	2.00	23.00	1.00	.00	.00	5.00	2.00	4.00
27.00	1165.00	1.00	1.00	2.00	.00	.00	5.00	2.00	5.00
28.00	1167.00	1.00	1.00	1.00	1.00	3.00	1.00	2.00	5.00
29.00	1169.00	2.00	25.00	2.00	.00	.00	5.00	2.00	5.00
30.00	1177.00	2.00	1.00	2.00	.00	.00	5.00	2.00	5.00
31.00	1183.00	2.00	1.00	2.00	.00	.00	5.00	2.00	5.00
32.00	1157.00	2.00	26.00	1.00	1.00	2.00	3.00	2.00	2.00
33.00	1191.00	1.00	1.00	2.00	.00	.00	5.00	2.00	5.00
34.00	1186.00	1.00	1.00	1.00	.00	.00	2.00	2.00	5.00
35.00	1174.00	2.00	13.00	2.00	.00	.00	5.00	2.00	1.00
36.00	1200.00	1.00	1.00	2.00	.00	.00	-1.00	2.00	5.00
37.00	1199.00	2.00	1.00	2.00	.00	.00	5.00	2.00	3.00
38.00	1202.00	1.00	35.00	2.00	.00	.00	5.00	2.00	3.00
39.00	1206.00	1.00	1.00	2.00	.00	.00	5.00	2.00	1.00
41.00	1219.00	2.00	4.00	1.00	.00	.00	-1.00	2.00	1.00
42.00	1210.00	1.00	1.00	2.00	.00	.00	-1.00	2.00	5.00
43.00	1214.00	1.00	1.00	2.00	.00	.00	5.00	2.00	5.00
44.00	1215.00	1.00	1.00	2.00	.00	.00	5.00	2.00	1.00
45.00	1218.00	2.00	1.00	2.00	.00	.00	5.00	2.00	1.00
46.00	1222.00	1.00	1.00	2.00	.00	.00	5.00	2.00	1.00
47.00	1224.00	1.00	18.00	1.00	1.00	2.00	6.00	1.00	.00
48.00	1213.00	2.00	4.00	1.00	1.00	2.00	2.00	2.00	1.00
49.00	1225.00	2.00	1.00	2.00	.00	.00	3.00	2.00	5.00
50.00	1226.00	1.00	1.00	1.00	1.00	2.00	3.00	2.00	1.00
51.00	1211.00	1.00	35.00	2.00	1.00	2.00	5.00	1.00	.00
52.00	1216.00	1.00	1.00	2.00	.00	.00	5.00	2.00	1.00
53.00	1244.00	1.00	18.00	2.00	1.00	2.00	6.00	2.00	4.00
54.00	1245.00	1.00	1.00	2.00	1.00	2.00	3.00	2.00	1.00
55.00	1250.00	1.00	1.00	1.00	.00	.00	5.00	2.00	1.00
56.00	1228.00	1.00	1.00	1.00	1.00	2.00	2.00	2.00	1.00

57.00	1237.00	1.00	4.00	2.00	.00	.00	3.00	2.00	1.00
58.00	1260.00	2.00	1.00	1.00	1.00	3.00	1.00	2.00	1.00
59.00	1253.00	1.00	1.00	1.00	1.00	2.00	6.00	2.00	1.00
60.00	1258.00	1.00	1.00	2.00	.00	.00	5.00	2.00	.00
61.00	1262.00	2.00	1.00	1.00	1.00	3.00	1.00	2.00	1.00
62.00	1268.00	2.00	1.00	2.00	.00	.00	5.00	2.00	5.00
63.00	1276.00	1.00	45.00	1.00	.00	.00	2.00	1.00	.00
64.00	1267.00	1.00	1.00	2.00	.00	.00	5.00	2.00	5.00
65.00	1266.00	1.00	1.00	2.00	.00	.00	5.00	2.00	5.00
66.00	1278.00	1.00	1.00	2.00	.00	.00	5.00	2.00	5.00
67.00	1279.00	2.00	11.00	1.00	1.00	1.00	6.00	2.00	1.00
68.00	1287.00	1.00	1.00	2.00	.00	.00	5.00	2.00	5.00
69.00	1291.00	2.00	1.00	2.00	.00	.00	5.00	2.00	5.00
70.00	1290.00	2.00	60.00	2.00	.00	.00	3.00	2.00	7.00
71.00	1300.00	1.00	18.00	2.00	1.00	2.00	6.00	2.00	4.00
72.00	1301.00	2.00	1.00	1.00	1.00	2.00	3.00	2.00	5.00
73.00	1270.00	2.00	1.00	2.00	1.00	1.00	5.00	2.00	1.00
74.00	-1.00	2.00	1.00	1.00	1.00	2.00	2.00	2.00	1.00
75.00	1304.00	2.00	75.00	2.00	.00	.00	5.00	2.00	4.00
76.00	1302.00	1.00	1.00	2.00	.00	.00	5.00	2.00	1.00
77.00	-1.00	1.00	1.00	1.00	1.00	2.00	2.00	2.00	1.00
78.00	1328.00	2.00	28.00	1.00	1.00	3.00	1.00	1.00	.00
79.00	1306.00	1.00	1.00	2.00	.00	.00	3.00	2.00	5.00
80.00	1333.00	1.00	1.00	2.00	.00	.00	3.00	2.00	5.00
81.00	1334.00	2.00	4.00	2.00	.00	.00	-1.00	2.00	1.00
82.00	1336.00	2.00	1.00	1.00	1.00	3.00	2.00	2.00	5.00
83.00	1340.00	2.00	40.00	2.00	1.00	1.00	3.00	2.00	4.00
84.00	1349.00	2.00	1.00	1.00	1.00	2.00	2.00	2.00	5.00
85.00	1344.00	1.00	38.00	1.00	1.00	3.00	1.00	1.00	.00
86.00	1344.00	1.00	38.00	1.00	1.00	3.00	1.00	1.00	
87.00	1357.00	1.00	8.00	1.00	.00	.00	5.00	2.00	1.00
88.00	1342.00	1.00	7.00	1.00	.00	.00	5.00	2.00	1.00
89.00	1356.00	1.00	12.00	2.00	1.00	1.00	6.00	2.00	1.00
90.00	1358.00	2.00	1.00	2.00	.00	.00	3.00	2.00	1.00
91.00	1362.00	2.00	54.00	1.00	1.00	2.00	2.00	1.00	.00
92.00	1364.00	1.00	21.00	2.00	.00	.00	2.00	2.00	1.00
93.00	1367.00	2.00	28.00	2.00	.00	.00	5.00	2.00	4.00
94.00	1371.00	1.00	1.00	1.00	1.00	3.00	1.00	2.00	5.00
95.00	1372.00	1.00	2.00	2.00	.00	.00	5.00	2.00	1.00
96.00	1376.00	2.00	5.00	1.00	1.00	1.00	1.00	1.00	.00
97.00	1377.00	2.00	28.00	1.00	.00	.00	3.00	2.00	4.00
98.00	1359.00	2.00	1.00	1.00	1.00	3.00	1.00	2.00	5.00
99.00	1378.00	2.00	15.00	2.00	1.00	1.00	6.00	1.00	.00
100.00	1380.00	1.00	2.00	2.00	.00	.00	3.00	2.00	5.00
101.00		1.00	1.00	1.00	1.00	2.00	2.00	2.00	1.00
102.00		2.00	12.00	2.00	1.00	1.00	6.00	2.00	1.00
103.00		1.00	60.00	2.00	1.00	2.00	2.00	2.00	4.00
104.00		2.00	25.00	2.00	.00	.00	3.00	2.00	2.00
105.00		2.00	1.00	1.00	.00	.00	3.00	2.00	1.00
106.00		2.00	1.00	2.00	.00	.00	5.00	2.00	5.00
107.00		1.00	1.00	1.00	.00	.00	5.00	2.00	1.00
108.00		1.00	1.00	2.00	.00	.00	5.00	2.00	1.00
109.00		2.00	1.00	1.00	1.00	2.00	2.00	2.00	1.00
110.00		2.00	1.00	1.00	1.00	3.00	1.00	2.00	1.00
111.00		2.00	1.00	2.00	.00	.00	5.00	2.00	1.00
112.00		1.00	1.00	2.00	.00	.00	5.00	2.00	5.00

113.00	1.00	1.00	2.00	.00	.00	5.00	2.00	1.00
114.00	2.00	6.00	1.00	.00	.00	5.00	2.00	1.00
115.00	2.00	1.00	1.00	1.00	3.00	2.00	2.00	1.00
116.00	2.00	24.00	1.00	1.00	2.00	3.00	2.00	2.00
117.00	1.00	1.00	2.00	.00	.00	5.00	2.00	5.00
118.00	1.00	1.00	1.00	.00	.00	5.00	2.00	5.00
119.00	1.00	42.00	2.00	.00	.00	5.00	1.00	.00
120.00	1.00	45.00	2.00	.00	.00	5.00	1.00	.00
121.00	1.00	1.00	2.00	.00	.00	5.00	2.00	1.00
122.00	1.00	11.00	2.00	1.00	1.00	6.00	2.00	1.00
123.00	1.00	22.00	1.00	1.00	2.00	6.00	2.00	3.00
124.00	2.00	1.00	1.00	1.00	3.00	1.00	1.00	.00
125.00	1.00	1.00	2.00	.00	.00	5.00	2.00	5.00
126.00	2.00	1.00	2.00	.00	.00	5.00	2.00	5.00
127.00	1.00	53.00	1.00	1.00	2.00	2.00	1.00	.00
128.00	1.00	1.00	2.00	.00	.00	5.00	2.00	5.00
129.00	1.00	1.00	2.00	.00	.00	5.00	2.00	5.00
130.00	2.00	1.00	2.00	.00	.00	5.00	2.00	1.00
131.00	2.00	45.00	2.00	.00	.00	5.00	2.00	4.00
132.00	2.00	1.00	1.00	.00	.00	5.00	2.00	1.00
133.00	1.00	1.00	1.00	.00	.00	5.00	2.00	5.00
134.00	1.00	58.00	2.00	.00	.00	5.00	1.00	.00
135.00	1.00	1.00	1.00	.00	.00	-1.00	2.00	5.00
136.00	2.00	25.00	2.00	.00	.00	3.00	2.00	2.00
137.00	2.00	25.00	2.00	.00	.00	3.00	2.00	2.00
138.00	2.00	1.00	2.00	.00	.00	5.00	2.00	1.00
139.00	1.00	28.00	1.00	1.00	3.00	1.00	1.00	.00
140.00	2.00	1.00	2.00	.00	.00	5.00	2.00	1.00
141.00	2.00	25.00	1.00	1.00	3.00	1.00	1.00	.00
142.00	1.00	1.00	2.00	.00	.00	5.00	2.00	5.00
143.00	2.00	1.00	2.00	.00	.00	5.00	2.00	5.00
144.00	2.00	1.00	2.00	.00	.00	5.00	2.00	1.00
145.00	1.00	1.00	2.00	.00	.00	5.00	2.00	5.00
146.00	1.00	2.00	1.00	1.00	3.00	1.00	2.00	1.00
147.00	1.00	1.00	2.00	.00	.00	5.00	2.00	5.00
148.00	2.00	30.00	2.00	.00	.00	5.00	2.00	4.00
149.00	1.00	1.00	2.00	.00	.00	5.00	2.00	5.00
150.00	2.00	63.00	1.00	.00	.00	3.00	2.00	7.00
151.00	1.00	1.00	2.00	.00	.00	5.00	2.00	5.00
152.00	1.00	32.00	1.00	1.00	3.00	1.00	1.00	.00
153.00	2.00	3.00	1.00	.00	.00	3.00	2.00	1.00
154.00	2.00	1.00	1.00	.00	.00	3.00	2.00	1.00
155.00	1.00	1.00	2.00	.00	.00	5.00	2.00	5.00
156.00	1.00	1.00	2.00	.00	.00	5.00	2.00	5.00
157.00	1.00	1.00	2.00	.00	.00	5.00	2.00	5.00
158.00	1.00	1.00	2.00	.00	.00	5.00	2.00	1.00
159.00	1.00	1.00	1.00	.00	.00	5.00	2.00	1.00
160.00	1.00	3.00	1.00	.00	.00	-1.00	2.00	1.00
161.00	1.00	1.00	2.00	.00	.00	5.00	2.00	1.00
162.00	1.00	75.00	2.00	.00	.00	5.00	2.00	5.00
163.00	2.00	23.00	1.00	1.00	3.00	1.00	2.00	7.00
164.00	1.00	52.00	2.00	.00	.00	7.00	2.00	5.00
165.00	1.00	1.00	2.00	.00	.00	7.00	2.00	5.00
166.00	1.00	23.00	1.00	1.00	2.00	7.00	1.00	.00
167.00	2.00	1.00	2.00	.00	.00	7.00	2.00	5.00
168.00	1.00	25.00	1.00	1.00	2.00	5.00	2.00	4.00

169.00	1.00	1.00	2.00	.00	.00	7.00	2.00	1.00
170.00	2.00	1.00	1.00	.00	.00	7.00	2.00	1.00
171.00	1.00	1.00	2.00	.00	.00	7.00	2.00	1.00
172.00	1.00	1.00	2.00	.00	.00	7.00	2.00	5.00
173.00	2.00	1.00	2.00	.00	.00	7.00	2.00	5.00
174.00	1.00	1.00	1.00	.00	.00	7.00	2.00	5.00
175.00	1.00	1.00	2.00	.00	.00	7.00	2.00	1.00
176.00	1.00	1.00	2.00	.00	.00	7.00	2.00	5.00
177.00	1.00	1.00	2.00	.00	.00	7.00	2.00	5.00
178.00	1.00	1.00	2.00	.00	.00	7.00	2.00	5.00
179.00	2.00	1.00	2.00	.00	.00	7.00	2.00	5.00
180.00	1.00	1.00	2.00	.00	.00	7.00	2.00	5.00
181.00	1.00	1.00	2.00	.00	.00	7.00	2.00	1.00
182.00	1.00	1.00	1.00	.00	.00	7.00	2.00	1.00
183.00	1.00	1.00	1.00	.00	.00	3.00	2.00	1.00
184.00	1.00	1.00	2.00	.00	.00	5.00	2.00	1.00
185.00	1.00	1.00	2.00	.00	.00	7.00	2.00	1.00
186.00	1.00	1.00	2.00	.00	.00	7.00	2.00	1.00
187.00	1.00	73.00	2.00	.00	.00	5.00	2.00	4.00
188.00	1.00	1.00	1.00	1.00	1.00	7.00	2.00	1.00
189.00	1.00	1.00	2.00	.00	.00	7.00	2.00	5.00
190.00	1.00	3.00	2.00	.00	.00	7.00	2.00	1.00
191.00	2.00	23.00	1.00	1.00	3.00	1.00	1.00	.00
192.00	1.00	1.00	2.00	1.00	1.00	7.00	2.00	1.00
193.00	1.00	18.00	1.00	1.00	2.00	6.00	2.00	3.00
194.00	1.00	1.00	2.00	.00	.00	-1.00	2.00	1.00
195.00	2.00	1.00	1.00	.00	.00	7.00	2.00	5.00
196.00	1.00	30.00	2.00	.00	.00	5.00	2.00	4.00
197.00	1.00	1.00	1.00	1.00	3.00	1.00	2.00	5.00
198.00	2.00	1.00	1.00	1.00	3.00	6.00	2.00	5.00
199.00	2.00	20.00	1.00	1.00	2.00	6.00	1.00	.00
200.00	1.00	1.00	2.00	.00	.00	7.00	2.00	1.00
201.00	1.00	1.00	2.00	.00	.00	7.00	2.00	1.00
202.00	2.00	1.00	2.00	.00	.00	7.00	2.00	1.00
203.00	1.00	37.00	1.00	1.00	3.00	1.00	1.00	.00
204.00	2.00	1.00	2.00	.00	.00	7.00	2.00	5.00
205.00	1.00	1.00	2.00	.00	.00	7.00	2.00	1.00
207.00	2.00	1.00	1.00	1.00	2.00	3.00	2.00	1.00
208.00	2.00	18.00	2.00	1.00	3.00	6.00	1.00	.00
209.00	2.00	1.00	2.00	.00	.00	7.00	2.00	1.00
210.00	1.00	1.00	1.00	.00	.00	-1.00	2.00	1.00
211.00	1.00	1.00	1.00	.00	.00	7.00	2.00	1.00
212.00	2.00	1.00	1.00	.00	.00	7.00	2.00	1.00
213.00	1.00	1.00	2.00	.00	.00	7.00	2.00	1.00
214.00	2.00	28.00	1.00	1.00	3.00	1.00	1.00	.00
215.00	1.00	1.00	2.00	.00	.00	3.00	2.00	1.00
216.00	1.00	1.00	2.00	.00	.00	7.00	2.00	5.00
217.00	1.00	9.00	2.00	.00	.00	7.00	2.00	1.00
218.00	1.00	1.00	2.00	.00	.00	7.00	2.00	5.00
219.00	2.00	5.00	2.00	.00	.00	7.00	2.00	1.00
220.00	1.00	1.00	2.00	.00	.00	7.00	2.00	1.00
221.00	1.00	1.00	1.00	.00	.00	7.00	2.00	5.00
222.00	1.00	1.00	1.00	.00	.00	7.00	2.00	1.00
223.00	1.00	1.00	2.00	.00	.00	7.00	2.00	5.00
224.00	2.00	1.00	2.00	.00	.00	7.00	2.00	1.00
225.00	1.00	1.00	2.00	.00	.00	7.00	2.00	1.00

226.00		1.00	1.00	2.00	.00	.00	7.00	2.00	1.00
227.00		2.00	7.00	2.00	1.00	2.00	5.00	2.00	1.00
228.00		2.00	1.00	2.00	.00	.00	3.00	2.00	5.00
229.00		2.00	1.00	2.00	.00	.00	7.00	2.00	5.00
230.00		2.00	1.00	2.00	.00	.00	7.00	2.00	1.00
231.00		2.00	23.00	1.00	1.00	3.00	1.00	1.00	.00
232.00		1.00	1.00	2.00	.00	.00	7.00	2.00	1.00
233.00		1.00	1.00	1.00	.00	.00	7.00	2.00	1.00
234.00		2.00	1.00	2.00	.00	.00	7.00	2.00	1.00
235.00		2.00	1.00	2.00	.00	.00	5.00	2.00	1.00
236.00		1.00	1.00	1.00	1.00	3.00	1.00	2.00	5.00
237.00		1.00	1.00	2.00	.00	.00	5.00	2.00	1.00
238.00		1.00	1.00	1.00	.00	.00	7.00	2.00	1.00
239.00		2.00	1.00	1.00	.00	.00	7.00	2.00	1.00
240.00	816.00	2.00	1.00	1.00	.00	.00	7.00	2.00	5.00
241.00	828.00	1.00	1.00	2.00	.00	.00	7.00	2.00	6.00
242.00	829.00	1.00	1.00	2.00	.00	.00	7.00	2.00	5.00
243.00	833.00	1.00	1.00	1.00	.00	.00	7.00	2.00	5.00
244.00	847.00	1.00	10.00	1.00	1.00	1.00	6.00	2.00	1.00
245.00	871.00	1.00	26.00	1.00	1.00	3.00	1.00	1.00	.00
246.00	859.00	2.00	28.00	1.00	.00	.00	3.00	2.00	2.00
247.00	889.00	1.00	60.00	2.00	.00	.00	5.00	2.00	5.00
248.00	952.00	1.00	18.00	2.00	1.00	2.00	6.00	2.00	3.00
249.00	974.00	2.00	26.00	2.00	.00	.00	3.00	2.00	3.00
250.00	1034.00	2.00	20.00	2.00	.00	.00	3.00	2.00	4.00
251.00	1042.00	2.00	27.00	2.00	.00	.00	3.00	2.00	2.00
252.00	1051.00	1.00	10.00	1.00	1.00	1.00	6.00	2.00	1.00
253.00	1055.00	2.00	37.00	2.00	.00	.00	5.00	2.00	2.00
254.00	1066.00	2.00	32.00	2.00	.00	.00	5.00	2.00	2.00
255.00	1103.00	1.00	14.00	1.00	1.00	1.00	6.00	2.00	1.00
256.00	1081.00	2.00	28.00	1.00	1.00	2.00	3.00	2.00	2.00
257.00		2.00	22.00	1.00	1.00	1.00	6.00	2.00	2.00
258.00	1116.00	1.00	8.00	1.00	1.00	1.00	6.00	2.00	1.00
259.00	1112.00	1.00	45.00	2.00	.00	.00	5.00	2.00	4.00
260.00	1157.00	1.00	21.00	2.00	1.00	2.00	6.00	2.00	4.00
261.00	1174.00	2.00	20.00	1.00	1.00	2.00	6.00	1.00	.00
262.00	1155.00	2.00	35.00	2.00	.00	.00	3.00	2.00	3.00
263.00	1193.00	2.00	27.00	2.00	1.00	2.00	3.00	2.00	2.00
264.00	1202.00	2.00	9.00	2.00	.00	.00	7.00	2.00	1.00
265.00	1199.00	1.00	20.00	2.00	.00	.00	5.00	2.00	4.00
1.00		2.00	1.00	2.00	.00	.00	5.00	2.00	1.00
2.00		2.00	4.00	2.00	.00	.00		2.00	1.00
3.00		1.00	14.00	2.00	.00	3.00	5.00	2.00	1.00
4.00		2.00	1.00	2.00	.00	.00	5.00	2.00	1.00
5.00		2.00	20.00	1.00	1.00	3.00	1.00	1.00	.00
6.00		2.00	30.00	1.00	1.00	3.00	1.00	1.00	.00
7.00		2.00	25.00	2.00	.00	.00	5.00	2.00	4.00
8.00		2.00	17.00	2.00	.00	.00	5.00	2.00	4.00
9.00		1.00	9.00	1.00	1.00	1.00	6.00	2.00	1.00
10.00		2.00	10.00	2.00	1.00	1.00	6.00	2.00	1.00
11.00		1.00	25.00	2.00	1.00	2.00	5.00	1.00	7.00
12.00		2.00	9.00	1.00	1.00	1.00	6.00	2.00	1.00
13.00		2.00	12.00	1.00	1.00	1.00	6.00	2.00	1.00
14.00		2.00	12.00	2.00	.00	.00	5.00	2.00	5.00
15.00		2.00	2.00	2.00	.00	.00	5.00	2.00	5.00
16.00		2.00	26.00	1.00	1.00	2.00	-1.00	1.00	.00

17.00	1.00	1.00	2.00	.00	.00	5.00	2.00	5.00
18.00	1.00	13.00	2.00	1.00	1.00	6.00	2.00	4.00
19.00	1.00	24.00	1.00	1.00	3.00	1.00	1.00	.00
20.00	2.00	30.00	1.00	1.00	3.00	1.00	1.00	.00
21.00	2.00	1.00	1.00	1.00	3.00	1.00	2.00	1.00
22.00	2.00	7.00	2.00	1.00	1.00	6.00	2.00	1.00
23.00	2.00	14.00	2.00	1.00	1.00	6.00	2.00	1.00
24.00	2.00	30.00	2.00	.00	.00	3.00	1.00	.00
25.00	1.00	25.00	1.00	1.00	3.00	1.00	1.00	.00
26.00	1.00	1.00	1.00	1.00	2.00	1.00	2.00	1.00
27.00	1.00	28.00	1.00	1.00	3.00	1.00	2.00	4.00
28.00	2.00	38.00	1.00	1.00	3.00	2.00	1.00	.00
29.00	1.00	1.00	1.00	.00	.00	-1.00	2.00	1.00
30.00	2.00	4.00	1.00	.00	.00	-1.00	2.00	1.00
31.00	1.00	1.00	1.00	.00	.00		2.00	1.00
32.00	2.00	32.00	2.00	.00	.00	3.00	1.00	.00
33.00	2.00	23.00	2.00	.00	.00	3.00	1.00	.00
34.00	2.00	32.00	2.00	.00	.00	3.00	1.00	.00
35.00	2.00	14.00	1.00	1.00	1.00	6.00	2.00	1.00
36.00	2.00	23.00	1.00	1.00	3.00	6.00	1.00	.00
37.00	2.00	23.00	1.00	1.00	3.00	6.00	1.00	.00
38.00	2.00	23.00	1.00	1.00	3.00	6.00	1.00	.00
39.00	1.00	1.00	1.00	.00	.00		2.00	1.00
40.00	2.00	2.00	2.00	.00	.00		2.00	1.00
41.00	2.00	10.00	1.00	1.00	1.00	6.00	2.00	1.00
42.00	1.00	10.00	2.00	.00	.00	5.00	2.00	1.00
43.00	1.00	1.00	1.00	.00	.00		2.00	1.00
44.00	1.00	1.00	2.00	.00	.00		2.00	5.00
45.00	1.00	15.00	1.00	1.00	3.00	1.00	1.00	.00
46.00	2.00	30.00	2.00	.00	.00	3.00	2.00	4.00
47.00	2.00	35.00	1.00	1.00	3.00	1.00	1.00	.00
48.00	2.00	1.00	1.00	1.00	3.00	2.00	1.00	.00
49.00	1.00	13.00	2.00	1.00	1.00	6.00	2.00	1.00
50.00	2.00	2.00	1.00	1.00	3.00	1.00	1.00	.00
51.00	2.00	28.00	1.00	1.00	3.00	2.00	1.00	.00
52.00	2.00	28.00	1.00	1.00	3.00	2.00	1.00	.00
53.00	2.00	28.00	1.00	1.00	3.00	1.00	1.00	.00
54.00	1.00	1.00	2.00	.00	.00	3.00	2.00	5.00
55.00	2.00	35.00	1.00	1.00	3.00	1.00	2.00	4.00
56.00	2.00	27.00	2.00	.00	.00	3.00	2.00	4.00
57.00	1.00	18.00	2.00	1.00	2.00	6.00	2.00	4.00
58.00	2.00	21.00	2.00	.00	.00	3.00	2.00	4.00
59.00	2.00	23.00	2.00	.00	.00	3.00	2.00	4.00
60.00	1.00	30.00	1.00	1.00	3.00	1.00	2.00	4.00
61.00	2.00	8.00	1.00	.00	.00	3.00	2.00	5.00
62.00	1.00	13.00	2.00	1.00	1.00	6.00	2.00	1.00
63.00	2.00	86.00	1.00	.00	.00	3.00	2.00	4.00
64.00	2.00	20.00	2.00	.00	.00	3.00	2.00	4.00
65.00	1.00	30.00	2.00	.00	.00	2.00	2.00	4.00
66.00	1.00	4.00	1.00	1.00	3.00	1.00	1.00	.00
67.00	1.00	1.00	1.00	.00	.00	3.00	2.00	1.00
68.00	1.00	30.00	2.00	1.00	3.00	1.00	1.00	.00
69.00	1.00	30.00	1.00	1.00	3.00	1.00	2.00	3.00
70.00	1.00	8.00	2.00	.00	.00	5.00	2.00	1.00
71.00	2.00	2.00	2.00	.00	.00	5.00	2.00	1.00
72.00	2.00	2.00	2.00	.00	.00	5.00	2.00	1.00

73.00	2.00	11.00	2.00	1.00	2.00	2.00	2.00	1.00
74.00	2.00	10.00	2.00	.00	.00	5.00	2.00	1.00
75.00	2.00	1.00	1.00	1.00	2.00	2.00	2.00	1.00
76.00	1.00	20.00	1.00	1.00	2.00	1.00	1.00	.00
77.00	2.00	1.00	1.00	1.00	3.00	1.00	2.00	1.00
78.00	1.00	8.00	1.00	1.00	1.00	6.00	2.00	1.00
79.00	2.00	1.00	1.00	1.00	2.00	2.00	2.00	1.00
80.00	1.00	1.00	1.00	1.00	1.00	1.00	2.00	1.00
81.00	2.00	4.00	1.00	1.00	3.00	1.00	2.00	1.00
82.00	2.00	1.00	2.00	.00	.00	5.00	2.00	1.00
83.00	2.00	2.00	1.00	1.00	3.00	1.00	2.00	1.00
84.00	2.00	20.00	1.00	1.00	2.00	2.00	1.00	.00
85.00	1.00	18.00	2.00	1.00	2.00	4.00	2.00	4.00
86.00	1.00	28.00	2.00	1.00	1.00	5.00	2.00	4.00
87.00	2.00	12.00	1.00	1.00	1.00	6.00	2.00	1.00
88.00	1.00	1.00	1.00	1.00	2.00	2.00	2.00	1.00
89.00	2.00	21.00	1.00	1.00	3.00	6.00	2.00	4.00
90.00	1.00	1.00	1.00	1.00	3.00	2.00	2.00	1.00
91.00	1.00	20.00	1.00	1.00	1.00	4.00	2.00	4.00
92.00	2.00	1.00	1.00	1.00	2.00	2.00	2.00	1.00
93.00	2.00	2.00	1.00	1.00	2.00	3.00	2.00	1.00
94.00	1.00	1.00	1.00	1.00	3.00	1.00	2.00	1.00
95.00	1.00	1.00	1.00	1.00	3.00	1.00	2.00	1.00
96.00	1.00	3.00	2.00	1.00	2.00	5.00	2.00	1.00
97.00	1.00	1.00	1.00	1.00	2.00	2.00	2.00	1.00
98.00	1.00	8.00	1.00	1.00	1.00	6.00	2.00	1.00
99.00	1.00	37.00	1.00	1.00	2.00	4.00	2.00	4.00
100.00	1.00	18.00	2.00	1.00	2.00	6.00	2.00	4.00
101.00	1.00	68.00	2.00	.00	.00	5.00	2.00	4.00
102.00	2.00	1.00	1.00	1.00	2.00	2.00	2.00	5.00
104.00	1.00	30.00	1.00	1.00	3.00	1.00	2.00	4.00
105.00	2.00	1.00	2.00	.00	.00	3.00	2.00	1.00
106.00	1.00	4.00	1.00	1.00	3.00	1.00	2.00	1.00
107.00	1.00	15.00	2.00	1.00	1.00	6.00	2.00	1.00
108.00	1.00	1.00	1.00	1.00	3.00	1.00	2.00	1.00
109.00	1.00	1.00	1.00	.00	.00	3.00	2.00	1.00
110.00	2.00	1.00	2.00	.00	.00	5.00	2.00	1.00
111.00	2.00	1.00	2.00	1.00	2.00	2.00	2.00	5.00
112.00	1.00	1.00	2.00	.00	.00	5.00	2.00	5.00
113.00	2.00	8.00	2.00	.00	.00	5.00	2.00	1.00
114.00	1.00	35.00	1.00	1.00	3.00	1.00	2.00	4.00
115.00	2.00	1.00	1.00	1.00	3.00	1.00	2.00	1.00
116.00	1.00	4.00	1.00	1.00	3.00	1.00	2.00	5.00
117.00	1.00	1.00	2.00	.00	.00	5.00	2.00	1.00
118.00	2.00	1.00	2.00	.00	.00	5.00	2.00	1.00
119.00	1.00	27.00	1.00	1.00	2.00	4.00	2.00	1.00
120.00	1.00	42.00	2.00	.00	.00	-1.00	2.00	4.00
121.00	1.00	13.00	2.00	1.00	1.00	6.00	2.00	1.00
122.00	2.00	1.00	1.00	1.00	3.00	1.00	2.00	1.00
123.00	1.00	22.00	2.00	.00	.00	5.00	2.00	4.00
124.00	2.00	11.00	2.00	1.00	1.00	6.00	1.00	.00
125.00	2.00	11.00	2.00	1.00	1.00	6.00	2.00	1.00
126.00	2.00	5.00	1.00	1.00	3.00	7.00	2.00	1.00
127.00	2.00	1.00	2.00	.00	.00	5.00	2.00	1.00
128.00	1.00	22.00	1.00	1.00	2.00	6.00	2.00	4.00
129.00	1.00	10.00	1.00	1.00	1.00	6.00	2.00	1.00

130.00	1.00	8.00	2.00	1.00	1.00	6.00	2.00	1.00
131.00	2.00	13.00	1.00	1.00	1.00	6.00	1.00	.00
132.00	1.00	49.00	1.00	1.00	3.00	2.00	1.00	.00
133.00	2.00	15.00	2.00	1.00	1.00	6.00	2.00	1.00
134.00	1.00	13.00	1.00	1.00	1.00	6.00	2.00	1.00
135.00	1.00	28.00	2.00	1.00	2.00	5.00	2.00	4.00
136.00	1.00	19.00	2.00	1.00	2.00	6.00	2.00	3.00
137.00	1.00	19.00	2.00	1.00	2.00	6.00	2.00	4.00
138.00	1.00	4.75	2.00	.00	.00	-1.00	2.00	1.00
139.00	1.00	10.00	2.00	1.00	1.00	6.00	2.00	1.00
140.00	2.00	35.00	1.00	1.00	2.00	3.00	1.00	.00
141.00	2.00	12.00	2.00	1.00	1.00	6.00	2.00	1.00
142.00	1.00	20.00	2.00	1.00	2.00	6.00	2.00	4.00
143.00	1.00	1.00	2.00	.00	.00	5.00	2.00	5.00
144.00	2.00	30.00	2.00	1.00	2.00	3.00	2.00	4.00
145.00	2.00	25.00	1.00	1.00	3.00	3.00	2.00	2.00
146.00	1.00	40.00	1.00	.00	.00	2.00	2.00	4.00
147.00	1.00	1.00	2.00	.00	.00	5.00	2.00	1.00
148.00	1.00	40.00	1.00	1.00	3.00	1.00	1.00	.00
149.00	1.00	1.00	1.00	.00	.00	4.00	2.00	1.00
150.00	2.00	21.00	2.00	1.00	2.00	6.00	2.00	4.00
151.00	2.00	30.00	2.00	1.00	1.00	5.00	2.00	4.00
151.00	1.00	1.00	2.00	.00	.00	5.00	1.00	.00
152.00	2.00	60.00	2.00	.00	.00	5.00	2.00	4.00
153.00	2.00	28.00	2.00	1.00	2.00	3.00	2.00	2.00
154.00	1.00	23.00	2.00	1.00	2.00	6.00	2.00	7.00
155.00	2.00	5.00	2.00	.00	.00	5.00	2.00	1.00
156.00	1.00	12.00	2.00	1.00	1.00	6.00	2.00	1.00
157.00	2.00	20.00	2.00	1.00	2.00	2.00	2.00	4.00
158.00	1.00	48.00	1.00	1.00	3.00	1.00	1.00	.00
159.00	2.00	4.00	2.00	.00	.00	5.00	2.00	3.00
160.00	2.00	30.00	1.00	1.00	2.00	3.00	2.00	4.00
161.00	2.00	20.00	1.00	1.00	2.00	3.00	2.00	2.00
162.00	1.00	18.00	2.00	1.00	2.00	6.00	2.00	4.00
163.00	1.00	4.00	1.00	1.00	3.00	2.00	2.00	1.00
164.00	1.00	36.00	2.00	.00	.00	5.00	2.00	5.00
165.00	1.00	28.00	2.00	.00	.00	5.00	2.00	4.00
166.00	1.00	25.00	1.00	1.00	3.00	1.00	2.00	4.00
167.00	1.00	4.00	1.00	.00	.00	-1.00	2.00	1.00
168.00	1.00	1.00	2.00	.00	.00	5.00	2.00	1.00
169.00	1.00	7.00	2.00	1.00	1.00	6.00	2.00	1.00
170.00	2.00	28.00	2.00	.00	.00	3.00	2.00	4.00
171.00	1.00	30.00	1.00	1.00	3.00	1.00	1.00	.00
172.00	2.00	1.00	2.00	.00	.00	-1.00	2.00	1.00
173.00	2.00	20.00	1.00	1.00	2.00	2.00	2.00	2.00
174.00	2.00	45.00	2.00	.00	.00	3.00	2.00	4.00
175.00	1.00	28.00	1.00	1.00	3.00	1.00	2.00	4.00
176.00	2.00	4.00	2.00	.00	.00	-1.00	2.00	3.00
177.00	2.00	3.00	1.00	.00	.00	-1.00	2.00	6.00
178.00	2.00	25.00	2.00	.00	.00	3.00	2.00	4.00
179.00	2.00	26.00	1.00	1.00	3.00	1.00	2.00	4.00
180.00	2.00	24.00	2.00	.00	.00	3.00	2.00	2.00
181.00	1.00	1.00	2.00	.00	.00	5.00	2.00	1.00
182.00	1.00	5.00	2.00	.00	.00	3.00	2.00	1.00
183.00	2.00	41.00	1.00	1.00	.00	1.00	1.00	.00
184.00	1.00	25.00	2.00	.00	.00	5.00	2.00	4.00

185.00		1.00	16.00	1.00	1.00	2.00	6.00	2.00	4.00
186.00		2.00	32.00	1.00	1.00	3.00	1.00	1.00	.00
187.00		2.00	9.00	1.00	1.00	1.00	6.00	2.00	1.00
188.00		1.00	1.00	1.00	1.00	3.00	1.00	2.00	1.00
189.00		2.00	18.00	2.00	1.00	2.00	6.00	2.00	4.00
190.00		2.00	20.00	2.00	.00	.00	3.00	2.00	4.00
191.00		2.00	1.00	2.00	.00	.00	5.00	2.00	1.00
192.00		2.00	28.00	1.00	1.00	3.00	1.00	2.00	4.00
193.00		2.00	20.00	2.00	.00	.00	5.00	2.00	4.00
194.00	355.00	1.00	65.00	2.00	1.00	1.00	5.00	2.00	4.00
195.00	349.00	1.00	17.00	2.00	1.00	2.00	6.00	2.00	4.00
196.00	352.00	1.00	4.00	1.00	.00	.00	7.00	2.00	1.00
197.00	360.00	1.00	4.00	1.00	.00	.00	7.00	2.00	1.00
198.00	342.00	1.00	16.00	2.00	1.00	1.00	6.00	2.00	1.00
199.00	334.00	2.00	20.00	1.00	1.00	2.00	6.00	2.00	4.00
200.00	335.00	2.00	23.00	1.00	1.00	3.00	1.00	2.00	7.00
201.00	392.00	1.00	1.00	1.00	.00	.00	7.00	2.00	5.00
202.00	364.00	1.00	3.00	1.00	.00	.00	7.00	2.00	1.00
203.00	369.00	1.00	23.00	2.00	1.00	3.00	1.00	2.00	7.00
204.00	372.00	1.00	32.00	2.00	1.00	2.00	3.00	2.00	4.00
205.00	377.00	1.00	1.00	1.00	.00	.00	7.00	2.00	5.00
206.00	388.00	1.00	50.00	2.00	.00	.00	5.00	2.00	5.00
207.00	415.00	1.00	12.00	2.00	.00	.00	7.00	2.00	1.00
208.00	397.00	2.00	40.00	2.00	.00	.00	5.00	2.00	4.00
209.00	396.00	1.00	25.00	2.00	1.00	3.00	1.00	2.00	4.00
210.00	426.00	1.00	27.00	2.00	1.00	2.00	2.00	1.00	.00
211.00	425.00	2.00	21.00	1.00	1.00	2.00	6.00	1.00	.00
212.00	344.00	2.00	50.00	1.00	.00	.00	3.00	1.00	.00
213.00	410.00	1.00	60.00	2.00	1.00	2.00	5.00	2.00	4.00
214.00	443.00	2.00	24.00	1.00	1.00	3.00	1.00	1.00	.00
215.00	456.00	2.00	1.00	2.00	.00	.00	7.00	1.00	.00
216.00	461.00	2.00	3.00	1.00	.00	.00	7.00	2.00	1.00
217.00	452.00	1.00	24.00	1.00	1.00	3.00	1.00	2.00	4.00
218.00	468.00	1.00	14.00	1.00	1.00	1.00	6.00	1.00	.00
219.00	471.00	1.00	45.00	1.00	1.00	3.00	1.00	1.00	.00
220.00	455.00	1.00	2.00	1.00	.00	.00	7.00	2.00	1.00
221.00	466.00	1.00	14.00	2.00	1.00	1.00	6.00	1.00	7.00
222.00	462.00	1.00	50.00	2.00	.00	.00	5.00	2.00	4.00
223.00	774.00	2.00	28.00	2.00	.00	.00	3.00	2.00	5.00
224.00	484.00	2.00	4.00	2.00	.00	.00	7.00	2.00	1.00
225.00	488.00	2.00	38.00	2.00	.00	.00	3.00	2.00	4.00
226.00	504.00	2.00	28.00	1.00	1.00	3.00	1.00	1.00	7.00
227.00	507.00	1.00	35.00	2.00	.00	.00	5.00	2.00	4.00
228.00	532.00	2.00	3.00	1.00	.00	.00	7.00	2.00	1.00
229.00	524.00	2.00	22.00	1.00	1.00	3.00	1.00	1.00	.00
230.00	533.00	2.00	8.00	1.00	1.00	1.00	6.00	2.00	1.00
231.00	511.00	1.00	1.00	2.00	.00	.00	.00	2.00	1.00
232.00	512.00	1.00	12.00	2.00	.00	.00	7.00	2.00	1.00
233.00	513.00	1.00	12.00	2.00	1.00	1.00	6.00	2.00	1.00
234.00	514.00	1.00	35.00	2.00	.00	.00	5.00	2.00	5.00
235.00	518.00	1.00	26.00	2.00	.00	.00	5.00	2.00	4.00
236.00	530.00	1.00	18.00	2.00	1.00	2.00	6.00	2.00	7.00
237.00	531.00	2.00	8.00	2.00	1.00	1.00	6.00	2.00	1.00
238.00	536.00	1.00	1.00	2.00	.00	.00	7.00	2.00	5.00
239.00	538.00	1.00	28.00	2.00	.00	.00	5.00	2.00	4.00
240.00	567.00	1.00	30.00	2.00	1.00	2.00	6.00	2.00	4.00

242.00	547.00	1.00	25.00	2.00	.00	.00	5.00	2.00	4.00
241.00	568.00	2.00	17.00	2.00	.00	.00	3.00	2.00	2.00
243.00	550.00	2.00	12.00	1.00	1.00	1.00	6.00	2.00	1.00
244.00	553.00	1.00	3.00	2.00	.00	.00	.00	2.00	1.00
245.00	559.00	2.00	2.00	2.00	.00	.00	.00	2.00	1.00
246.00	562.00	2.00	60.00	2.00	.00	.00	3.00	2.00	4.00
247.00	563.00	1.00	25.00	2.00	.00	.00	3.00	2.00	2.00
248.00	564.00	1.00	56.00	1.00	1.00	2.00	2.00	2.00	4.00
249.00	576.00	2.00	20.00	2.00	1.00	2.00	6.00	2.00	5.00
250.00	580.00	1.00	17.00	2.00	1.00	3.00	6.00	2.00	1.00
251.00	581.00	1.00	43.00	2.00	1.00	3.00	1.00	2.00	7.00
252.00	598.00	1.00	65.00	2.00	.00	.00	5.00	1.00	7.00
253.00	596.00	1.00	12.00	2.00	1.00	1.00	.00	2.00	1.00
254.00	593.00	2.00	50.00	2.00	.00	.00	3.00	2.00	7.00
255.00	619.00	2.00	12.00	1.00	1.00	1.00	.00	2.00	1.00
256.00	620.00	1.00	4.00	2.00	.00	.00	.00	2.00	1.00
257.00	622.00	1.00	29.00	1.00	1.00	3.00	1.00	2.00	4.00
258.00	630.00	1.00	9.00	2.00	1.00	1.00	.00	2.00	1.00
259.00	634.00	2.00	12.00	2.00	1.00	1.00	.00	2.00	1.00
260.00	638.00	2.00	3.00	2.00	.00	.00	.00	2.00	1.00
261.00	644.00	2.00	28.00	2.00	.00	.00	3.00	2.00	2.00
262.00	649.00	1.00	1.00	1.00	.00	.00	.00	2.00	1.00
263.00	652.00	2.00	14.00	1.00	1.00	2.00	.00	2.00	1.00
264.00	656.00	2.00	3.00	1.00	.00	.00	.00	2.00	1.00
265.00	667.00	1.00	18.00	2.00	.00	.00	5.00	2.00	7.00
266.00	668.00	2.00	17.00	2.00	.00	.00	4.00	2.00	4.00
267.00	671.00	2.00	7.00	1.00	.00	.00	7.00	2.00	1.00
268.00	673.00	1.00	30.00	1.00	1.00	3.00	2.00	2.00	7.00
269.00	674.00	2.00	40.00	2.00	1.00	2.00	3.00	2.00	4.00

sample	organism	ampicillin	Erythromycin	CEF	oxacillin	pencillin	Cli	AMC	SXT	Ttc
2.00	1.00	1.00		3.00				1.00	1.00	.00
2.00	1.00									
2.00	1.00									
2.00	1.00	1.00		3.00				1.00	1.00	.00
2.00	3.00	1.00		3.00				1.00	1.00	.00
2.00	1.00	1.00		3.00				1.00	1.00	.00
2.00	4.00	1.00		3.00				1.00	.00	.00
2.00	5.00	1.00		3.00				1.00	1.00	1.00
2.00	6.00	1.00		3.00				1.00	1.00	1.00
2.00	5.00									
2.00	2.00	1.00		3.00				1.00	1.00	1.00
1.00	2.00	1.00		3.00				1.00	1.00	1.00
2.00	7.00	1.00	3.00		3.00	3.00	1.00		1.00	1.00
2.00	5.00	1.00		3.00				1.00	1.00	.00
1.00	6.00	1.00		3.00				1.00	1.00	1.00
2.00	8.00									
2.00	8.00									
2.00	9.00									
2.00	2.00	1.00		2.00				1.00	1.00	1.00
2.00	8.00		3.00		3.00	3.00	1.00		1.00	1.00
2.00	8.00		3.00		3.00	3.00	1.00		1.00	1.00
2.00	10.00	1.00		3.00				1.00	1.00	1.00
2.00	5.00	1.00						1.00	1.00	1.00
2.00	9.00		3.00		3.00	3.00	3.00		1.00	1.00
2.00	5.00	1.00		3.00				1.00	1.00	1.00
1.00	6.00	1.00		3.00				1.00	1.00	1.00
2.00	5.00	1.00		3.00				1.00	1.00	1.00
2.00	11.00	1.00						1.00	1.00	1.00
2.00	7.00									
2.00	12.00	1.00		3.00				1.00	1.00	1.00
2.00	5.00	1.00	3.00					1.00	1.00	1.00
2.00	8.00		3.00		3.00	3.00	1.00		1.00	1.00
2.00	5.00	1.00					-1.00	1.00	1.00	1.00
2.00	8.00		3.00		3.00	3.00	3.00		1.00	1.00
2.00	8.00									
2.00	11.00	1.00						1.00	1.00	1.00
2.00	5.00	1.00						1.00	1.00	1.00
2.00	7.00									
2.00	5.00	1.00						1.00	1.00	1.00
1.00	5.00	1.00						1.00	1.00	1.00
2.00	12.00	1.00						1.00	1.00	1.00
2.00	8.00		3.00		3.00	3.00	3.00		1.00	1.00
2.00	8.00		3.00		3.00	3.00	3.00		1.00	1.00
2.00	14.00	1.00						.00	1.00	1.00
2.00	14.00	1.00		3.00				1.00	1.00	1.00
3.00	7.00	1.00	3.00		3.00	3.00	1.00		.00	1.00
2.00	9.00									
2.00	14.00	1.00						1.00	1.00	1.00
2.00	8.00									
2.00	8.00									
2.00	8.00									
2.00	7.00									
2.00	14.00	1.00						1.00	1.00	1.00
2.00	8.00									
2.00	7.00		3.00		3.00	3.00	3.00		1.00	1.00

2.00	8.00									
2.00	14.00	1.00						1.00	.00	.00
2.00	8.00									
2.00	12.00	1.00						1.00	1.00	1.00
2.00	15.00									
2.00	5.00	1.00						1.00	1.00	1.00
3.00	16.00	1.00						1.00	1.00	1.00
2.00	12.00	1.00						1.00	1.00	1.00
2.00	8.00									
2.00	5.00	1.00						1.00	1.00	1.00
2.00	17.00		3.00		3.00	3.00	3.00		1.00	1.00
2.00	5.00	1.00						1.00	1.00	1.00
2.00	5.00	1.00						1.00	1.00	1.00
2.00	8.00		3.00		3.00	3.00	1.00		1.00	1.00
2.00	7.00		3.00		3.00	3.00	3.00		1.00	.00
2.00	5.00	1.00						1.00	1.00	1.00
2.00	8.00									
2.00	18.00	1.00					3.00	1.00	1.00	.00
2.00	7.00									
2.00	19.00									
2.00	20.00	1.00						1.00	1.00	.00
1.00	14.00	1.00		3.00				1.00	.00	.00
2.00	7.00		3.00		3.00	1.00	1.00		1.00	1.00
2.00	11.00	1.00		3.00				1.00	1.00	1.00
2.00	7.00									
2.00	14.00	1.00						1.00	1.00	1.00
2.00	8.00									
5.00	7.00									
4.00	5.00	1.00						1.00	1.00	1.00
4.00	7.00		3.00		3.00	3.00	3.00		1.00	1.00
1.00	12.00	1.00						1.00	.00	1.00
2.00	21.00	1.00						1.00	1.00	1.00
2.00	22.00		3.00		3.00	3.00	3.00		1.00	1.00
2.00	23.00									
1.00	5.00									
2.00	2.00	1.00		3.00				1.00	1.00	1.00
2.00	22.00		3.00		3.00	3.00	3.00		1.00	.00
2.00	5.00	1.00		3.00				1.00	1.00	1.00
2.00	1.00									
1.00	7.00		3.00		3.00	3.00	1.00		1.00	1.00
2.00	24.00	.00	3.00		3.00	3.00	3.00		1.00	1.00
2.00	25.00		3.00		3.00	3.00	3.00		.00	1.00
5.00	26.00									
2.00	5.00	1.00		3.00				1.00	1.00	1.00
2.00	22.00		3.00		3.00	3.00	3.00		1.00	1.00
1.00	2.00	1.00		3.00				1.00	1.00	1.00
2.00	30.00	1.00	3.00		3.00		3.00		1.00	.00
1.00	5.00	1.00		3.00				1.00	1.00	1.00
2.00	8.00		1.00		3.00		3.00		.00	.00
2.00	5.00	1.00		3.00				1.00	1.00	1.00
2.00	8.00		3.00		3.00	3.00	3.00		1.00	1.00
2.00	20.00									
2.00	8.00									
2.00	5.00									
2.00	1.00									
2.00	6.00	1.00		3.00				1.00	1.00	1.00

2.00	5.00	1.00		3.00				1.00	1.00	1.00
1.00	6.00									
2.00	20.00									
1.00	5.00	1.00		3.00				.00	1.00	1.00
2.00	7.00									
2.00	1.00									
3.00	7.00									
3.00	7.00									
5.00	7.00									
2.00	19.00									
2.00	7.00									
1.00	6.00	1.00		3.00				1.00	1.00	1.00
2.00	5.00	1.00		3.00				1.00	1.00	1.00
2.00	5.00	1.00		3.00				.00	1.00	1.00
1.00	6.00									
2.00	5.00	1.00		3.00				1.00	1.00	1.00
2.00	5.00	1.00		3.00				1.00	1.00	1.00
2.00	5.00									
2.00	19.00									
2.00	7.00									
2.00	7.00									
2.00	15.00	1.00						1.00	1.00	1.00
2.00	5.00	1.00		3.00				1.00	1.00	1.00
1.00	1.00	1.00		3.00				1.00	.00	.00
1.00	32.00									
2.00	1.00									
4.00	12.00									
2.00	5.00	1.00						.00	1.00	1.00
1.00	6.00									
2.00	5.00	1.00		3.00				1.00	1.00	1.00
2.00	5.00									
2.00	5.00	1.00		3.00				1.00	1.00	1.00
2.00	2.00	1.00		3.00				1.00	1.00	1.00
1.00	6.00	1.00		3.00				1.00	1.00	1.00
2.00	7.00									
2.00	11.00									
2.00	5.00	1.00		3.00				1.00	1.00	1.00
1.00	19.00									
2.00	5.00	1.00		3.00				.00	1.00	1.00
4.00	7.00									
1.00	3.00	1.00		3.00				.00	1.00	1.00
1.00	6.00	1.00		1.00				.00	1.00	1.00
2.00	5.00	1.00		3.00				1.00	1.00	1.00
2.00	1.00	1.00						1.00	1.00	1.00
2.00	5.00	1.00		3.00				.00	1.00	1.00
2.00	5.00	1.00		3.00				1.00	1.00	1.00
2.00	2.00	1.00		3.00				1.00	1.00	1.00
2.00	5.00	1.00		3.00				1.00	1.00	.00
1.00	11.00	1.00		3.00				1.00	1.00	1.00
3.00	12.00									
1.00	19.00									
2.00	5.00	1.00						1.00	1.00	1.00
2.00	6.00	1.00						1.00	1.00	1.00
4.00	20.00									
2.00	5.00	1.00						1.00	1.00	1.00
2.00	7.00		1.00		3.00	3.00	3.00		1.00	1.00

2.00	5.00	1.00					1.00	1.00	1.00
2.00	5.00	1.00					1.00	1.00	1.00
2.00	5.00								
2.00	5.00	1.00					1.00	1.00	1.00
2.00	5.00	1.00					1.00	1.00	1.00
2.00	5.00	1.00					1.00	1.00	1.00
2.00	5.00	1.00					1.00	1.00	1.00
2.00	5.00	1.00					1.00	1.00	1.00
2.00	5.00	1.00					1.00	1.00	1.00
2.00	5.00	1.00					1.00	1.00	1.00
2.00	5.00	1.00					1.00	1.00	1.00
2.00	5.00	1.00					1.00	1.00	1.00
2.00	5.00	1.00					1.00	1.00	1.00
2.00	6.00	1.00					1.00	1.00	1.00
2.00	19.00		3.00	3.00	3.00	3.00		1.00	1.00
2.00	5.00	1.00					1.00	1.00	1.00
2.00	5.00	1.00					1.00	1.00	1.00
2.00	5.00								
2.00	7.00								
2.00	5.00	1.00					1.00	1.00	1.00
2.00	2.00	1.00					1.00	1.00	1.00
1.00	5.00	1.00					1.00	1.00	1.00
1.00	7.00		3.00	3.00	3.00	3.00		1.00	1.00
2.00	6.00	1.00					1.00	1.00	.00
5.00	6.00	1.00					1.00	1.00	.00
2.00	6.00	1.00					.00	1.00	1.00
2.00	5.00	1.00					1.00	1.00	1.00
1.00	2.00	1.00					1.00	1.00	1.00
2.00	1.00	1.00					1.00	.00	.00
2.00	5.00	1.00					1.00	1.00	.00
2.00	5.00	1.00					1.00	1.00	1.00
1.00	6.00	1.00					1.00	1.00	1.00
2.00	7.00								
2.00	1.00	1.00					1.00	.00	1.00
2.00	6.00	1.00					1.00	1.00	1.00
2.00	5.00	1.00					1.00	.00	1.00
2.00	6.00	1.00					1.00	1.00	1.00
1.00	17.00		3.00	3.00	3.00	3.00		1.00	1.00
1.00	11.00	1.00					1.00	1.00	1.00
2.00	5.00	1.00					1.00	1.00	1.00
2.00	5.00	1.00					1.00	1.00	1.00
3.00	7.00								
2.00	5.00	1.00					1.00	1.00	1.00
2.00	5.00	1.00					1.00	1.00	1.00
1.00	6.00								
1.00	2.00	1.00					1.00	1.00	.00
2.00	7.00		3.00	3.00	3.00	1.00		1.00	1.00
2.00	14.00	1.00					1.00	1.00	.00
2.00	20.00	1.00					1.00	1.00	1.00
1.00	28.00	1.00					1.00	1.00	1.00
2.00	5.00	1.00					.00	1.00	.00
2.00	14.00	1.00					1.00	1.00	.00
2.00	11.00	1.00					1.00	1.00	1.00
2.00	1.00	1.00					1.00	.00	.00
2.00	5.00	1.00					1.00	1.00	1.00
2.00	14.00	1.00					1.00	1.00	1.00

5.00	7.00								
5.00	7.00								
2.00	11.00	1.00					1.00	1.00	1.00
2.00	5.00	1.00					1.00	1.00	1.00
2.00	5.00	1.00					1.00	1.00	1.00
1.00	18.00	1.00					1.00	1.00	1.00
2.00	5.00	1.00					1.00	1.00	1.00
2.00	5.00	1.00					1.00	1.00	1.00
2.00	12.00	1.00					1.00	1.00	1.00
2.00	11.00								
2.00	5.00	1.00					1.00	1.00	1.00
2.00	5.00	1.00					1.00	1.00	1.00
2.00	5.00								
2.00	12.00								
2.00	5.00								
2.00	12.00								
2.00	5.00	1.00					1.00	1.00	1.00
2.00	5.00								
2.00	5.00								
8.00	30.00								
2.00	31.00	1.00					1.00	1.00	1.00
2.00	2.00								
3.00	5.00	1.00					1.00	1.00	1.00
3.00	7.00								
2.00	7.00		3.00	3.00	3.00	3.00		1.00	1.00
2.00	6.00	1.00					1.00	1.00	1.00
2.00	7.00		3.00	3.00	3.00	3.00		1.00	1.00
2.00	19.00		3.00	3.00	3.00	3.00		1.00	1.00
2.00	6.00	1.00					.00	1.00	1.00
1.00	5.00	1.00					1.00	1.00	1.00
2.00	7.00		3.00	3.00		3.00		1.00	1.00
2.00	6.00	1.00					1.00	1.00	1.00
2.00	31.00	1.00					1.00	1.00	1.00
2.00	5.00	1.00					.00	1.00	1.00
2.00	6.00	1.00					.00	1.00	1.00
3.00	7.00		3.00			3.00		.00	.00
3.00	1.00								
2.00	7.00								
2.00	9.00		3.00	3.00	1.00	3.00		1.00	.00
2.00	7.00		3.00	3.00	3.00	3.00		1.00	.00
2.00	.00								
1.00	.00								
2.00	.00								
7.00	.00								
8.00	.00								
7.00	.00								
2.00	.00								
2.00	.00								
7.00	.00								
7.00	.00								
2.00	.00								
2.00	.00								
2.00	.00								
2.00	.00								
2.00	.00								
2.00	.00								

2. 00	. 00
2. 00	. 00
1. 00	. 00
1. 00	. 00
2. 00	. 00
2. 00	. 00
2. 00	. 00
2. 00	. 00
7. 00	. 00
1. 00	. 00
2. 00	. 00
8. 00	. 00
7. 00	. 00
1. 00	. 00
7. 00	. 00
1. 00	. 00
9. 00	. 00
9. 00	. 00
1. 00	. 00
7. 00	. 00
1. 00	. 00
2. 00	. 00
7. 00	. 00
7. 00	. 00
2. 00	. 00
2. 00	. 00
2. 00	. 00
2. 00	. 00
2. 00	. 00
7. 00	. 00
1. 00	. 00
1. 00	. 00
2. 00	. 00
1. 00	. 00
1. 00	. 00
9. 00	. 00
1. 00	. 00
7. 00	. 00
2. 00	. 00
2. 00	. 00
2. 00	. 00
1. 00	. 00
2. 00	. 00
2. 00	. 00
7. 00	. 00
1. 00	. 00
2. 00	. 00
2. 00	. 00
7. 00	. 00
1. 00	. 00
7. 00	. 00
7. 00	. 00
2. 00	. 00
2. 00	. 00
2. 00	. 00
2. 00	. 00

2. 00	. 00
2. 00	. 00
2. 00	. 00
8. 00	. 00
2. 00	. 00
2. 00	. 00
2. 00	. 00
2. 00	. 00
1. 00	. 00
2. 00	. 00
7. 00	. 00
1. 00	. 00
2. 00	. 00
2. 00	. 00
1. 00	. 00
2. 00	. 00
2. 00	. 00
1. 00	. 00
2. 00	. 00
7. 00	. 00
1. 00	. 00
1. 00	. 00
7. 00	. 00
7. 00	. 00
7. 00	. 00
1. 00	. 00
2. 00	. 00
2. 00	. 00
2. 00	. 00
2. 00	. 00
2. 00	. 00
7. 00	. 00
2. 00	. 00
2. 00	. 00
2. 00	. 00
2. 00	. 00
2. 00	. 00
7. 00	. 00
2. 00	. 00
2. 00	. 00
2. 00	. 00
7. 00	. 00
7. 00	. 00
2. 00	. 00
7. 00	. 00
7. 00	. 00
7. 00	. 00
1. 00	. 00
1. 00	. 00
2. 00	. 00
1. 00	. 00
1. 00	. 00
7. 00	. 00
7. 00	. 00
2. 00	. 00
2. 00	. 00

7. 00	. 00
1. 00	. 00
7. 00	. 00
2. 00	. 00
2. 00	. 00
2. 00	. 00
2. 00	. 00
2. 00	. 00
2. 00	. 00
1. 00	. 00
9. 00	. 00
2. 00	. 00
6. 00	. 00
2. 00	. 00
2. 00	. 00
2. 00	. 00
2. 00	. 00
2. 00	. 00
2. 00	. 00
2. 00	. 00
2. 00	. 00
2. 00	. 00
2. 00	. 00
2. 00	. 00
2. 00	. 00
2. 00	. 00
2. 00	. 00
2. 00	. 00
2. 00	. 00
1. 00	. 00
2. 00	. 00
2. 00	. 00
2. 00	. 00
2. 00	. 00
2. 00	. 00
2. 00	. 00
2. 00	. 00
2. 00	. 00
2. 00	. 00
2. 00	. 00
2. 00	. 00
2. 00	. 00
2. 00	. 00
1. 00	. 00
2. 00	. 00
2. 00	. 00
2. 00	. 00
2. 00	. 00
5. 00	. 00
2. 00	. 00
2. 00	. 00
2. 00	. 00
2. 00	. 00
1. 00	. 00
1. 00	. 00
2. 00	. 00

2. 00	. 00
1. 00	. 00
2. 00	. 00
2. 00	. 00
2. 00	. 00
2. 00	. 00
2. 00	. 00
2. 00	. 00
2. 00	. 00
2. 00	. 00
2. 00	. 00
2. 00	. 00
2. 00	. 00
2. 00	. 00
2. 00	. 00
2. 00	. 00
2. 00	. 00
2. 00	. 00
2. 00	. 00
2. 00	. 00
2. 00	. 00
2. 00	. 00
1. 00	. 00
2. 00	. 00
2. 00	. 00
1. 00	. 00
1. 00	. 00
2. 00	. 00
2. 00	. 00
1. 00	. 00
1. 00	. 00
1. 00	. 00
2. 00	. 00
1. 00	. 00
1. 00	. 00
2. 00	. 00
2. 00	. 00
2. 00	. 00
2. 00	. 00
2. 00	. 00
2. 00	. 00
2. 00	. 00
2. 00	. 00
1. 00	. 00
1. 00	. 00
1. 00	. 00
2. 00	. 00
2. 00	. 00
2. 00	. 00
2. 00	. 00
2. 00	. 00
2. 00	. 00
2. 00	. 00
2. 00	. 00
2. 00	. 00
2. 00	. 00

1. 00	. 00
1. 00	. 00
2. 00	. 00
2. 00	. 00
2. 00	. 00
2. 00	. 00
2. 00	. 00
2. 00	. 00
2. 00	. 00
2. 00	. 00
2. 00	. 00
2. 00	. 00
2. 00	. 00
2. 00	. 00
2. 00	. 00
2. 00	. 00
2. 00	. 00
2. 00	. 00
2. 00	. 00
2. 00	. 00
2. 00	. 00
2. 00	. 00
2. 00	. 00
2. 00	. 00
2. 00	. 00
2. 00	. 00
2. 00	. 00
2. 00	. 00
2. 00	. 00

CIP	CAF	GEN	CFP	CXM	CRO	CXT	CAZ	ESBL	Bacteriatype
1.00	1.00	1.00	1.00	1.00	1.00	1.00	1.00	1.00	2.00
									2.00
									2.00
1.00	.00	1.00	1.00	1.00	1.00	1.00	1.00	1.00	2.00
.00	1.00	1.00	1.00	1.00	1.00	.00	1.00	.00	2.00
1.00	.00	1.00	1.00	1.00	1.00	1.00	1.00	1.00	2.00
.00	1.00	1.00	1.00	1.00	1.00	1.00	1.00	1.00	2.00
.00	1.00	1.00	1.00	1.00	1.00	.00	1.00	1.00	2.00
1.00	1.00	1.00	1.00	1.00	1.00	1.00	1.00	1.00	2.00
									2.00
1.00	1.00	1.00	1.00	1.00	1.00	1.00	1.00	1.00	2.00
1.00	1.00	1.00	1.00	1.00	1.00	1.00	1.00	1.00	2.00
1.00	1.00	1.00			1.00	1.00			1.00
.00	1.00	1.00	1.00	1.00	1.00	.00	1.00	1.00	2.00
1.00	1.00	1.00	1.00	1.00	1.00	1.00	1.00	1.00	2.00
									1.00
									1.00
									1.00
.00	1.00	1.00	1.00	1.00	1.00	1.00	1.00	1.00	2.00
.00	1.00	1.00			1.00	.00			1.00
1.00	1.00	1.00			1.00	1.00			1.00
.00	.00	1.00	1.00	1.00	1.00	.00	1.00	.00	2.00
1.00	1.00	1.00	1.00	1.00	1.00	1.00	1.00	1.00	2.00
1.00	1.00	1.00			1.00	1.00			1.00
.00	1.00	1.00	1.00	1.00	1.00	.00	1.00	1.00	2.00
1.00	.00	.00	1.00	1.00	1.00	1.00	1.00	1.00	2.00
1.00	1.00	1.00	1.00	1.00	1.00	1.00	1.00	1.00	2.00
1.00	1.00	1.00	1.00	1.00	1.00	1.00	1.00	1.00	2.00
									1.00
.00	.00	1.00	1.00	1.00	1.00	.00	1.00	1.00	2.00
1.00	1.00	1.00	1.00	1.00	1.00	1.00	1.00	1.00	2.00
1.00	.00	1.00			1.00	.00			1.00
1.00	1.00	1.00	1.00	1.00	1.00	.00	1.00	1.00	2.00
1.00	1.00	1.00			1.00	.00			1.00
									1.00
1.00	1.00	1.00	1.00	1.00	1.00	1.00	1.00	1.00	2.00
1.00	.00	1.00	1.00	1.00	1.00	.00	1.00	1.00	2.00
									1.00
1.00	1.00	1.00	1.00	1.00	1.00	1.00	1.00	1.00	2.00
1.00	1.00	.00	1.00	1.00	1.00	1.00	1.00	1.00	2.00
.00	1.00	1.00	1.00	.00	1.00	.00	1.00	1.00	2.00
.00	1.00	.00			1.00	1.00			1.00
1.00	.00	.00			1.00	.00			1.00
1.00	1.00	1.00	1.00	.00	1.00	.00	1.00	.00	2.00
1.00	.00	1.00	1.00	1.00	1.00	.00	1.00	1.00	2.00
.00	.00	.00			1.00	.00			1.00
									1.00
.00	.00	.00	1.00	1.00	1.00	.00	1.00	1.00	2.00
									1.00
									1.00
									1.00
									1.00
1.00	.00	.00	1.00	1.00	1.00	1.00	1.00	1.00	2.00
									1.00
.00	1.00	1.00			1.00	.00			1.00

									1.00
.00	.00	1.00	1.00	1.00	1.00	1.00	1.00	1.00	2.00
									1.00
1.00	1.00	1.00	1.00	1.00	1.00	.00	1.00	1.00	2.00
									2.00
1.00	1.00	1.00	1.00	1.00	1.00	.00	1.00	1.00	2.00
1.00	1.00	.00	1.00	.00	1.00	.00	.00	.00	2.00
1.00	1.00	1.00	1.00	1.00	1.00	.00	1.00	1.00	2.00
									1.00
1.00	.00	1.00	1.00	1.00	1.00	.00	1.00	1.00	2.00
.00	1.00	1.00			1.00	.00			1.00
1.00	1.00	1.00	1.00	1.00	1.00	1.00	1.00	1.00	2.00
.00	.00	1.00	1.00	1.00	1.00	.00	1.00	1.00	2.00
.00	.00	.00			.00	1.00			1.00
1.00	.00	1.00			.00	1.00			1.00
.00	.00	1.00	1.00	1.00	1.00	1.00	1.00	1.00	2.00
									1.00
1.00	.00	1.00	1.00	1.00	1.00	1.00	1.00	.00	2.00
									1.00
									1.00
1.00	1.00	1.00	1.00	1.00	1.00	1.00	1.00	1.00	2.00
.00	.00	.00	1.00	1.00	.00	.00	1.00	1.00	2.00
.00	.00	1.00			1.00	.00			1.00
1.00	1.00	1.00	1.00	1.00	1.00	.00	1.00	.00	2.00
									1.00
1.00	.00	1.00	1.00	1.00	1.00	.00	1.00	.00	2.00
									1.00
									1.00
1.00	1.00	1.00	1.00	1.00	1.00	1.00	1.00	1.00	2.00
1.00	1.00	1.00			1.00	1.00			1.00
.00	1.00	1.00	1.00	1.00	1.00	1.00	1.00	1.00	2.00
1.00	.00	1.00	1.00	1.00	1.00	1.00	1.00	.00	2.00
1.00	1.00	1.00			1.00	1.00			1.00
									1.00
									2.00
.00	.00	1.00	1.00	1.00	1.00	.00	1.00	.00	2.00
1.00	1.00	1.00			1.00	.00			1.00
1.00	1.00	1.00	1.00	1.00	1.00	1.00	1.00	1.00	2.00
									2.00
1.00	.00	.00			1.00	.00			1.00
.00	1.00	.00			.00	.00			1.00
.00	1.00	.00			1.00	.00			1.00
									2.00
1.00	.00	1.00	1.00	1.00	1.00	1.00	1.00	1.00	2.00
1.00	.00	1.00			1.00	1.00			1.00
.00	1.00	1.00	1.00	1.00	1.00	1.00	1.00	1.00	2.00
1.00	1.00	1.00			1.00	1.00			2.00
1.00	1.00	1.00	1.00	1.00	1.00	1.00	1.00	1.00	2.00
.00	1.00	1.00			1.00	1.00			1.00
1.00	.00	1.00	1.00	1.00	1.00	.00	1.00	.00	2.00
.00	1.00	.00			1.00	1.00			1.00
									2.00
									1.00
									2.00
									2.00
1.00	1.00	1.00	1.00	1.00	1.00	.00	1.00	1.00	2.00

.00	1.00	1.00	1.00	1.00	1.00	.00	1.00	1.00	2.00
									2.00
									2.00
.00	1.00	1.00	1.00	1.00	1.00	.00	1.00	1.00	2.00
									1.00
									2.00
									1.00
									1.00
									1.00
									1.00
									1.00
1.00	.00	1.00	1.00	1.00	1.00	1.00	1.00	1.00	2.00
1.00	1.00	1.00	1.00	1.00	1.00	1.00	1.00	1.00	2.00
.00	1.00	1.00	1.00	1.00	1.00	.00	1.00	1.00	2.00
									2.00
.00	1.00	1.00	1.00	1.00	1.00	.00	1.00	1.00	2.00
.00	1.00	1.00	1.00	1.00	1.00	.00	1.00	.00	2.00
									2.00
									1.00
									1.00
									1.00
1.00	1.00	.00	.00	.00	1.00	1.00	.00	1.00	2.00
.00	1.00	1.00	1.00	1.00	1.00	.00	1.00	1.00	2.00
.00	1.00	.00	1.00	1.00	1.00	1.00	1.00	.00	2.00
									2.00
									2.00
									2.00
.00	1.00	1.00	1.00	1.00	1.00	1.00	1.00	1.00	2.00
									2.00
.00	.00	1.00	1.00	1.00	1.00	1.00	1.00	1.00	2.00
									2.00
.00	1.00	1.00	1.00	1.00	1.00	.00	1.00	1.00	2.00
.00	1.00	1.00	1.00	1.00	1.00	.00	1.00	1.00	2.00
1.00	.00	.00	1.00	1.00	1.00	1.00	1.00	1.00	2.00
									1.00
									2.00
.00	.00	1.00	1.00	1.00	1.00	.00	1.00	1.00	2.00
									1.00
.00	1.00	1.00	1.00	1.00	1.00	.00	1.00	1.00	2.00
									1.00
.00	1.00	1.00	.00	.00	.00	.00	.00	.00	2.00
.00	1.00	1.00	.00	.00	.00	.00	1.00	.00	2.00
.00	1.00	1.00	1.00	1.00	1.00	.00	1.00	1.00	2.00
.00	1.00	1.00	1.00	1.00	1.00	1.00	1.00	1.00	2.00
.00	.00	1.00	1.00	1.00	.00	1.00	1.00	1.00	2.00
.00	1.00	1.00	1.00	1.00	1.00	.00	1.00	1.00	2.00
.00	1.00	1.00	1.00	1.00	1.00	.00	1.00	1.00	2.00
.00	.00	1.00	1.00	1.00	1.00	1.00	1.00	1.00	2.00
.00	1.00	1.00	1.00	1.00	1.00	1.00	1.00	1.00	2.00
									2.00
									1.00
.00	1.00	1.00	1.00	1.00	1.00	.00	1.00	1.00	2.00
.00	1.00	1.00	1.00	1.00	1.00	.00	1.00	1.00	2.00
									2.00
.00	1.00	1.00	1.00	1.00	1.00	.00	1.00	1.00	2.00
.00	.00	1.00			1.00	.00			1.00

.00	1.00	1.00	1.00	1.00	1.00	.00	1.00	1.00	2.00
.00	1.00	1.00	1.00	1.00	1.00	1.00	1.00	1.00	2.00
									2.00
.00	1.00	.00	1.00	.00	1.00	.00	1.00	.00	2.00
.00	1.00	1.00	1.00	1.00	1.00	1.00	1.00	1.00	2.00
.00	1.00	1.00	1.00	1.00	1.00	1.00	1.00	1.00	2.00
.00	1.00	1.00	1.00	1.00	1.00	.00	1.00	1.00	2.00
.00	.00	1.00	1.00	1.00	1.00	.00	1.00	1.00	2.00
.00	1.00	.00	1.00	1.00	1.00	1.00	1.00	1.00	2.00
.00	1.00	.00	1.00	1.00	1.00	.00	1.00	1.00	2.00
.00	1.00	1.00	1.00	1.00	1.00	.00	1.00	1.00	2.00
.00	1.00	1.00	1.00	1.00	1.00	1.00	1.00	1.00	2.00
.00	1.00	1.00	1.00	1.00	1.00	.00	1.00	1.00	2.00
1.00	.00	1.00	1.00	1.00	1.00	1.00	1.00	1.00	2.00
1.00	1.00	.00			1.00	.00			1.00
.00	1.00	1.00	1.00	1.00	1.00	.00	1.00	1.00	2.00
.00	1.00	1.00	1.00	1.00	1.00	.00	1.00	1.00	2.00
									2.00
									1.00
.00	.00	.00	1.00	1.00	1.00	.00	1.00	1.00	2.00
.00	1.00	1.00	1.00	1.00	1.00	.00	1.00	1.00	2.00
1.00	1.00	.00	1.00	1.00	1.00	.00	1.00	1.00	2.00
1.00	1.00	.00			1.00	.00			1.00
1.00	1.00	.00	1.00	1.00	1.00	.00	1.00	1.00	2.00
.00	1.00	.00	1.00	1.00	1.00	.00	1.00	1.00	2.00
.00	1.00	.00	.00	.00	1.00	.00	1.00	.00	2.00
.00	.00	.00	1.00	1.00	1.00	.00	1.00	1.00	2.00
.00	.00	.00	1.00	1.00	1.00	1.00	1.00	1.00	2.00
.00	.00	.00	1.00	1.00	1.00	1.00	1.00	1.00	2.00
.00	1.00	.00	1.00	1.00	1.00	.00	1.00	1.00	2.00
1.00	1.00	1.00	1.00	1.00	1.00	.00	1.00	1.00	2.00
1.00	1.00	.00	1.00	1.00	1.00	.00	1.00	1.00	2.00
									1.00
1.00	1.00	1.00	1.00	1.00	1.00	1.00	1.00	1.00	2.00
1.00	.00	.00	1.00	1.00	1.00	.00	1.00	1.00	2.00
.00	.00	.00	1.00	1.00	1.00	1.00	1.00	1.00	2.00
1.00	1.00	.00	1.00	1.00	1.00	1.00	1.00	1.00	2.00
1.00	1.00	1.00			1.00	.00			1.00
1.00	1.00	1.00	1.00	1.00	1.00	1.00	1.00	1.00	2.00
1.00	1.00	1.00	1.00	1.00	1.00	1.00	1.00	1.00	2.00
1.00	.00	1.00	1.00	1.00	1.00	.00	1.00	1.00	2.00
									1.00
.00	1.00	1.00	1.00	1.00	1.00	1.00	1.00	1.00	2.00
1.00	1.00	1.00	1.00	1.00	1.00	1.00	1.00	1.00	2.00
									2.00
.00	.00	1.00	1.00	1.00	1.00	.00	1.00	1.00	2.00
.00	.00	.00			1.00	1.00			1.00
1.00	1.00	1.00	1.00	1.00	1.00	1.00	1.00	1.00	2.00
.00	1.00	1.00	1.00	1.00	1.00	1.00	1.00	1.00	2.00
1.00	.00	1.00	1.00	1.00	1.00	1.00	1.00	1.00	2.00
1.00	.00	1.00	1.00	1.00	1.00	.00	1.00	1.00	2.00
1.00	1.00	1.00	1.00	1.00	1.00	.00	1.00	1.00	2.00
1.00	1.00	1.00	1.00	1.00	1.00	1.00	1.00	1.00	2.00
1.00	1.00	.00	1.00	1.00	1.00	1.00	1.00	.00	2.00
1.00	1.00	1.00	1.00	1.00	1.00	.00	1.00	1.00	2.00
.00	1.00	1.00	1.00	1.00	1.00	.00	1.00	1.00	2.00

									1.00
									1.00
1.00	1.00	.00	1.00	1.00	1.00	1.00	1.00	1.00	2.00
1.00	1.00	1.00	1.00	1.00	1.00	1.00	1.00	1.00	2.00
.00	.00	.00	1.00	1.00	1.00	1.00	1.00	1.00	2.00
.00	1.00	1.00	1.00	1.00	.00	.00	1.00	1.00	2.00
.00	1.00	1.00	1.00	1.00	1.00	1.00	1.00	1.00	2.00
1.00	1.00	1.00	1.00	1.00	1.00	1.00	1.00	1.00	2.00
1.00	1.00	1.00	1.00	1.00	1.00	1.00	1.00	1.00	2.00
									2.00
1.00	.00	1.00	1.00	1.00	1.00	.00	1.00	1.00	2.00
1.00	1.00	1.00	1.00	1.00	1.00	.00	1.00	1.00	2.00
									2.00
									2.00
									2.00
									2.00
1.00	1.00	1.00	1.00	1.00	1.00	1.00	1.00	1.00	2.00
									2.00
									2.00
									2.00
1.00	1.00	1.00	1.00	1.00	1.00	1.00	1.00	1.00	2.00
									2.00
.00	1.00	1.00	1.00	1.00	1.00	1.00	1.00	.00	2.00
									1.00
1.00	1.00	1.00			1.00	1.00			1.00
.00	.00	.00	.00	.00	.00	.00	.00	.00	2.00
1.00	.00	1.00			1.00	1.00			1.00
1.00	1.00	1.00			1.00	1.00			1.00
.00	.00	.00	.00	1.00	.00	.00	1.00	.00	2.00
.00	.00	1.00	1.00	1.00	1.00	1.00	1.00	1.00	2.00
.00	1.00	1.00			1.00	1.00			1.00
.00	1.00	1.00	1.00	1.00	1.00	1.00	1.00	1.00	2.00
.00	1.00	1.00	1.00	1.00	1.00	1.00	1.00	1.00	2.00
.00	.00	1.00	.00	.00	1.00	.00	.00	1.00	2.00
1.00	.00	1.00	1.00	1.00	1.00	1.00	1.00	.00	2.00
.00	1.00	1.00			.00	1.00			1.00
									2.00
									1.00
1.00	1.00	.00			1.00	.00			1.00
1.00	.00	.00			1.00	1.00			1.00

Bacterial_infection	Meropenum	merge_organism	merge1	MDR1	MDR2	R8	R9	R10	R11
1.00	.00	1.00	1.00	1.00	1.00	.00	.00	.00	1.00
1.00		1.00	1.00						
1.00		1.00	1.00						
1.00	.00	1.00	1.00	1.00	1.00	.00	.00	1.00	.00
1.00	.00	3.00	3.00	1.00	1.00	.00	1.00	.00	.00
1.00	1.00	1.00	1.00	1.00	1.00	.00	.00	1.00	.00
1.00	1.00	3.00	3.00	1.00	1.00	.00	1.00	.00	.00
1.00	.00	4.00	4.00	1.00	1.00	.00	.00	1.00	.00
1.00	.00	5.00	5.00	1.00	1.00	.00	.00	.00	.00
1.00		4.00	4.00						
1.00	.00	2.00	2.00	1.00	1.00	.00	.00	.00	.00
1.00	.00	2.00	2.00	1.00	1.00	.00	.00	.00	.00
1.00		6.00	6.00						
1.00	1.00	4.00	4.00	1.00	1.00	.00	1.00	.00	.00
1.00	.00	5.00	5.00	1.00	1.00	.00	.00	.00	.00
1.00		7.00	7.00						
1.00		7.00	7.00						
1.00		8.00	8.00						
1.00	.00	2.00	2.00	1.00	1.00	.00	.00	.00	1.00
1.00		7.00	7.00						
1.00		7.00	7.00						
1.00	.00	16.00	16.00	1.00	1.00	.00	1.00	.00	.00
1.00	.00	4.00	4.00	1.00	1.00	.00	.00	.00	.00
1.00		8.00	8.00						
1.00	.00	4.00	4.00	1.00	1.00	.00	.00	1.00	.00
1.00	.00	5.00	5.00	1.00	1.00	.00	.00	1.00	.00
1.00	1.00	4.00	4.00	1.00	1.00	.00	.00	.00	.00
1.00	.00	2.00	2.00	1.00	1.00	.00	.00	.00	.00
1.00		6.00	6.00						
1.00	.00	9.00	9.00	1.00	1.00	.00	1.00	.00	.00
1.00	1.00	4.00	4.00	1.00	1.00	.00	.00	.00	.00
1.00		7.00	7.00						
1.00	.00	4.00	4.00	1.00	1.00	.00	.00	.00	1.00
1.00		7.00	7.00						
1.00		7.00	7.00						
1.00	1.00	2.00	2.00	1.00	1.00	.00	.00	.00	.00
1.00	1.00	4.00	4.00	1.00	1.00	.00	.00	1.00	.00
1.00		6.00	6.00						
1.00	.00	4.00	4.00	1.00	1.00	.00	.00	.00	.00
1.00	.00	4.00	4.00	1.00	1.00	.00	.00	.00	1.00
1.00	.00	9.00	9.00	1.00	1.00	.00	1.00	.00	.00
1.00		7.00	7.00						
1.00		7.00	7.00						
1.00	.00	4.00	4.00	1.00	1.00	.00	1.00	.00	.00
1.00	1.00	4.00	4.00	1.00	1.00	.00	.00	1.00	.00
1.00		6.00	6.00						
1.00		8.00	8.00						
1.00	.00	4.00	4.00	1.00	1.00	1.00	.00	.00	.00
1.00		7.00	7.00						
1.00		7.00	7.00						
1.00		7.00	7.00						
1.00		6.00	6.00						
1.00	.00	4.00	4.00	1.00	1.00	.00	.00	1.00	.00
1.00		7.00	7.00						
1.00		6.00	6.00						

1.00		7.00	7.00						
1.00	.00	4.00	4.00	1.00	1.00	1.00	.00	.00	.00
1.00		7.00	7.00						
1.00	1.00	9.00	9.00	1.00	1.00	.00	.00	.00	1.00
1.00		4.00	4.00						
1.00	.00	4.00	4.00	1.00	1.00	.00	.00	.00	1.00
1.00	.00	10.00	10.00	1.00	1.00	1.00	.00	.00	.00
1.00	.00	9.00	9.00	1.00	1.00	.00	.00	.00	1.00
1.00		7.00	7.00						
1.00	.00	4.00	4.00	1.00	1.00	.00	.00	1.00	.00
1.00		8.00	8.00						
1.00	1.00	4.00	4.00	1.00	1.00	.00	.00	.00	.00
1.00	.00	4.00	4.00	1.00	1.00	.00	1.00	.00	.00
1.00		7.00	7.00						
1.00		6.00	6.00						
1.00	.00	4.00	4.00	1.00	1.00	.00	.00	1.00	.00
1.00		7.00	7.00						
1.00	.00	11.00	11.00	1.00	1.00	.00	.00	1.00	.00
1.00		6.00	6.00						
1.00		8.00	8.00						
1.00	.00	12.00	12.00	1.00	1.00	.00	.00	.00	1.00
1.00	.00	4.00	4.00	1.00	.00	.00	.00	.00	.00
1.00		6.00	6.00						
1.00	.00	2.00	2.00	1.00	1.00	.00	.00	.00	1.00
1.00		6.00	6.00						
1.00	.00	4.00	4.00	1.00	1.00	.00	.00	1.00	.00
1.00		7.00	7.00						
1.00		6.00	6.00						
1.00	.00	4.00	4.00	1.00	1.00	.00	.00	.00	.00
1.00		6.00	6.00						
1.00	.00	9.00	9.00	1.00	1.00	.00	.00	1.00	.00
1.00	.00	12.00	12.00	1.00	1.00	.00	.00	.00	1.00
1.00		7.00	7.00						
1.00		7.00	7.00						
1.00		4.00	4.00						
1.00	.00	2.00	2.00	1.00	1.00	.00	1.00	.00	.00
1.00		7.00	7.00						
1.00	.00	4.00	4.00	1.00	1.00	.00	.00	.00	.00
1.00		1.00	1.00						
1.00		6.00	6.00						
1.00		7.00	7.00						
1.00		7.00	7.00						
1.00		13.00	13.00						
1.00	.00	4.00	4.00	1.00	1.00	.00	.00	.00	1.00
1.00		7.00	7.00						
1.00	.00	2.00	2.00	1.00	1.00	.00	.00	.00	1.00
1.00		14.00	14.00						
1.00	.00	4.00	4.00	1.00	1.00	.00	.00	.00	.00
1.00		7.00	7.00						
1.00	.00	4.00	4.00	1.00	1.00	.00	.00	1.00	.00
1.00		7.00	7.00						
1.00		12.00	12.00						
1.00		7.00	7.00						
1.00		4.00	4.00						
1.00		1.00	1.00						
1.00	.00	5.00	5.00	1.00	1.00	.00	.00	.00	1.00

1.00	.00	4.00	4.00	1.00	1.00	.00	.00	1.00	.00
1.00		5.00	5.00						
1.00		12.00	12.00						
1.00	.00	4.00	4.00	1.00	1.00	.00	1.00	.00	.00
1.00		6.00	6.00						
1.00		1.00	1.00						
1.00		6.00	6.00						
1.00		6.00	6.00						
1.00		6.00	6.00						
1.00		8.00	8.00						
1.00		6.00	6.00						
1.00	.00	5.00	5.00	1.00	1.00	.00	.00	.00	1.00
1.00	1.00	4.00	4.00	1.00	1.00	.00	.00	.00	.00
1.00	.00	4.00	4.00	1.00	1.00	.00	1.00	.00	.00
1.00		5.00	5.00						
1.00	.00	4.00	4.00	1.00	1.00	.00	.00	1.00	.00
1.00	.00	4.00	4.00	1.00	1.00	.00	.00	1.00	.00
1.00		4.00	4.00						
1.00		8.00	8.00						
1.00		6.00	6.00						
1.00		6.00	6.00						
1.00	.00	4.00	4.00	1.00	1.00	1.00	.00	.00	.00
1.00	.00	4.00	4.00	1.00	1.00	.00	.00	1.00	.00
1.00	.00	1.00	1.00	1.00	1.00	1.00	.00	.00	.00
1.00		15.00	15.00						
1.00		1.00	1.00						
1.00		9.00	9.00						
1.00	.00	4.00	4.00	1.00	1.00	.00	.00	1.00	.00
1.00		5.00	5.00						
1.00	.00	4.00	4.00	1.00	1.00	.00	.00	1.00	.00
1.00		4.00	4.00						
1.00	.00	4.00	4.00	1.00	1.00	.00	.00	1.00	.00
1.00	.00	2.00	2.00	1.00	1.00	.00	.00	1.00	.00
1.00	.00	5.00	5.00	1.00	1.00	.00	.00	1.00	.00
1.00		6.00	6.00						
1.00		2.00	2.00						
1.00	1.00	4.00	4.00	1.00	1.00	.00	1.00	.00	.00
1.00		8.00	8.00						
1.00	.00	4.00	4.00	1.00	1.00	.00	1.00	.00	.00
1.00		6.00	6.00						
1.00	.00	3.00	3.00	1.00	1.00	.00	.00	.00	.00
1.00	.00	5.00	5.00	1.00	1.00	.00	.00	.00	.00
1.00	.00	4.00	4.00	1.00	1.00	.00	.00	1.00	.00
1.00	.00	1.00	1.00	1.00	1.00	.00	.00	.00	1.00
1.00	.00	4.00	4.00	1.00	1.00	1.00	.00	.00	.00
1.00	.00	4.00	4.00	1.00	1.00	.00	.00	1.00	.00
1.00	.00	2.00	2.00	1.00	1.00	.00	.00	1.00	.00
1.00	.00	4.00	4.00	1.00	1.00	.00	1.00	.00	.00
1.00	.00	2.00	2.00	1.00	1.00	.00	.00	.00	1.00
1.00		9.00	9.00						
1.00		8.00	8.00						
1.00	.00	4.00	4.00	1.00	1.00	.00	.00	1.00	.00
1.00	.00	5.00	5.00	1.00	1.00	.00	.00	1.00	.00
1.00		12.00	12.00						
1.00	.00	4.00	4.00	1.00	1.00	.00	.00	1.00	.00
1.00		6.00	6.00						

1.00	.00	4.00	4.00	1.00	1.00	.00	.00	1.00	.00
1.00	.00	4.00	4.00	1.00	1.00	.00	.00	.00	1.00
1.00		4.00	4.00						
1.00	.00	4.00	4.00	1.00	1.00	1.00	.00	.00	.00
1.00	1.00	4.00	4.00	1.00	1.00	.00	.00	.00	1.00
1.00	.00	4.00	4.00	1.00	1.00	.00	.00	.00	1.00
1.00	1.00	4.00	4.00	1.00	1.00	.00	.00	1.00	.00
1.00	.00	4.00	4.00	1.00	1.00	.00	1.00	.00	.00
1.00	.00	4.00	4.00	1.00	1.00	.00	.00	1.00	.00
1.00	.00	4.00	4.00	1.00	1.00	.00	1.00	.00	.00
1.00	.00	4.00	4.00	1.00	1.00	.00	.00	1.00	.00
1.00	.00	4.00	4.00	1.00	1.00	.00	.00	.00	1.00
1.00	.00	4.00	4.00	1.00	1.00	.00	.00	1.00	.00
1.00	.00	5.00	5.00	1.00	1.00	.00	.00	.00	1.00
1.00		8.00	8.00						
1.00	1.00	4.00	4.00	1.00	1.00	.00	.00	1.00	.00
1.00	1.00	4.00	4.00	1.00	1.00	.00	.00	1.00	.00
1.00		4.00	4.00						
1.00		6.00	6.00						
1.00	.00	4.00	4.00	1.00	1.00	1.00	.00	.00	.00
1.00	.00	2.00	2.00	1.00	1.00	.00	.00	1.00	.00
1.00	1.00	4.00	4.00	1.00	1.00	.00	.00	1.00	.00
1.00		6.00	6.00						
1.00	.00	5.00	5.00	1.00	1.00	.00	1.00	.00	.00
1.00	.00	5.00	5.00	1.00	1.00	1.00	.00	.00	.00
1.00	.00	5.00	5.00	1.00	1.00	.00	.00	.00	.00
1.00	.00	4.00	4.00	1.00	1.00	1.00	.00	.00	.00
1.00	.00	2.00	2.00	1.00	1.00	.00	1.00	.00	.00
1.00	.00	1.00	1.00	1.00	1.00	.00	.00	.00	.00
1.00	.00	4.00	4.00	1.00	1.00	1.00	.00	.00	.00
1.00	1.00	4.00	4.00	1.00	1.00	.00	.00	.00	1.00
1.00	1.00	5.00	5.00	1.00	1.00	.00	.00	1.00	.00
1.00		6.00	6.00						
1.00	.00	1.00	1.00	1.00	1.00	.00	.00	.00	1.00
1.00	.00	5.00	5.00	1.00	1.00	.00	1.00	.00	.00
1.00	.00	4.00	4.00	1.00	1.00	1.00	.00	.00	.00
1.00	1.00	5.00	5.00	1.00	1.00	.00	.00	.00	1.00
1.00		8.00	8.00						
1.00	.00	2.00	2.00	1.00	1.00	.00	.00	.00	.00
1.00	.00	4.00	4.00	1.00	1.00	.00	.00	.00	.00
1.00	.00	4.00	4.00	1.00	1.00	.00	.00	1.00	.00
1.00		6.00	6.00						
1.00	.00	4.00	4.00	1.00	1.00	.00	.00	.00	1.00
1.00	1.00	4.00	4.00	1.00	1.00	.00	.00	.00	.00
1.00		5.00	5.00						
1.00	.00	2.00	2.00	1.00	1.00	1.00	.00	.00	.00
1.00		6.00	6.00						
1.00	1.00	4.00	4.00	1.00	1.00	.00	.00	.00	1.00
1.00	.00	12.00	12.00	1.00	1.00	.00	.00	.00	1.00
1.00	.00	9.00	9.00	1.00	1.00	.00	.00	.00	1.00
1.00	.00	4.00	4.00	1.00	1.00	1.00	.00	.00	.00
1.00	.00	4.00	4.00	1.00	1.00	.00	.00	1.00	.00
1.00	1.00	2.00	2.00	1.00	1.00	.00	.00	.00	.00
1.00	.00	1.00	1.00	1.00	1.00	.00	1.00	.00	.00
1.00	.00	4.00	4.00	1.00	1.00	.00	.00	.00	1.00
1.00	.00	4.00	4.00	1.00	1.00	.00	.00	1.00	.00

1.00		6.00	6.00						
1.00		6.00	6.00						
1.00	1.00	2.00	2.00	1.00	1.00	.00	.00	.00	1.00
1.00	.00	4.00	4.00	1.00	1.00	.00	.00	.00	.00
1.00	.00	4.00	4.00	1.00	1.00	.00	1.00	.00	.00
1.00	.00	11.00	11.00	1.00	1.00	.00	1.00	.00	.00
1.00	.00	4.00	4.00	1.00	1.00	.00	.00	.00	1.00
1.00	.00	4.00	4.00	1.00	1.00	.00	.00	.00	.00
1.00	.00	9.00	9.00	1.00	1.00	.00	.00	.00	.00
1.00		2.00	2.00						
1.00	.00	4.00	4.00	1.00	1.00	.00	.00	1.00	.00
1.00	.00	4.00	4.00	1.00	1.00	.00	.00	.00	1.00
1.00		4.00	4.00						
1.00		9.00	9.00						
1.00		4.00	4.00						
1.00		9.00	9.00						
1.00	.00	4.00	4.00	1.00	1.00	.00	.00	.00	.00
1.00		4.00	4.00						
1.00		4.00	4.00						
1.00		14.00	14.00						
1.00	.00	4.00	4.00	1.00	1.00	.00	.00	.00	.00
1.00		2.00	2.00						
1.00	.00	4.00	4.00	1.00	1.00	.00	.00	.00	1.00
1.00		6.00	6.00						
1.00		6.00	6.00						
1.00	.00	5.00	5.00	1.00	1.00	.00	.00	.00	.00
1.00		6.00	6.00						
1.00		8.00	8.00						
1.00	.00	5.00	5.00	1.00	1.00	.00	.00	.00	.00
1.00	.00	4.00	4.00	1.00	1.00	.00	.00	1.00	.00
1.00		6.00	6.00						
1.00	.00	5.00	5.00	1.00	1.00	.00	.00	.00	1.00
1.00	.00	4.00	4.00	1.00	1.00	.00	.00	.00	1.00
1.00	.00	4.00	4.00	1.00	1.00	.00	.00	.00	.00
1.00	.00	5.00	5.00	1.00	1.00	.00	.00	1.00	.00
1.00		6.00	6.00						
1.00		1.00	1.00						
1.00		6.00	6.00						
1.00		8.00	8.00						
1.00		6.00	6.00						
.00									
.00									
.00									
.00									
.00									
.00									
.00									
.00									
.00									
.00									
.00									
.00									
.00									
.00									
.00									
.00									

. 00
. 00
. 00
. 00
. 00
. 00
. 00
. 00
. 00
. 00
. 00
. 00
. 00
. 00
. 00
. 00
. 00
. 00
. 00
. 00
. 00
. 00
. 00
. 00
. 00
. 00
. 00
. 00
. 00
. 00
. 00
. 00
. 00
. 00
. 00
. 00
. 00
. 00
. 00
. 00
. 00
. 00
. 00
. 00
. 00
. 00
. 00
. 00
. 00
. 00
. 00
. 00
. 00
. 00
. 00
. 00

. 00
. 00
. 00
. 00
. 00
. 00
. 00
. 00
. 00
. 00
. 00
. 00
. 00
. 00
. 00
. 00
. 00
. 00
. 00
. 00
. 00
. 00
. 00
. 00
. 00
. 00
. 00
. 00
. 00
. 00
. 00
. 00
. 00
. 00
. 00
. 00
. 00
. 00
. 00
. 00
. 00
. 00
. 00
. 00
. 00
. 00
. 00
. 00
. 00
. 00
. 00
. 00
. 00
. 00
. 00
. 00

. 00
. 00
. 00
. 00
. 00
. 00
. 00
. 00
. 00
. 00
. 00
. 00
. 00
. 00
. 00
. 00
. 00
. 00
. 00
. 00
. 00
. 00
. 00
. 00
. 00
. 00
. 00
. 00
. 00
. 00
. 00
. 00
. 00
. 00
. 00
. 00
. 00
. 00
. 00
. 00
. 00
. 00
. 00
. 00
. 00
. 00
. 00
. 00
. 00
. 00
. 00
. 00
. 00
. 00
. 00
. 00

.00
.00
.00
.00
.00
.00
.00
.00
.00
.00
.00
.00
.00
.00
.00
.00
.00
.00
.00
.00
.00
.00
.00
.00
.00
.00
.00
.00
.00
.00
.00
.00
.00
.00
.00
.00
.00
.00
.00
.00
.00
.00
.00
.00
.00
.00
.00
.00
.00
.00
.00
.00
.00
.00
.00
.00

. 00
. 00
. 00
. 00
. 00
. 00
. 00
. 00
. 00
. 00
. 00
. 00
. 00
. 00
. 00
. 00
. 00
. 00
. 00
. 00
. 00
. 00
. 00
. 00
. 00
. 00
. 00
. 00
. 00

R12	R0	R1	R2	R3	R4	R5	R6	R7	Age_group
.00	.00	.00	.00	.00	.00	.00	.00	.00	1.00
									1.00
									1.00
.00	.00	.00	.00	.00	.00	.00	.00	.00	1.00
.00	.00	.00	.00	.00	.00	.00	.00	.00	1.00
.00	.00	.00	.00	.00	.00	.00	.00	.00	1.00
.00	.00	.00	.00	.00	.00	.00	.00	.00	1.00
.00	.00	.00	.00	.00	.00	.00	.00	.00	1.00
1.00	.00	.00	.00	.00	.00	.00	.00	.00	1.00
									1.00
1.00	.00	.00	.00	.00	.00	.00	.00	.00	1.00
1.00	.00	.00	.00	.00	.00	.00	.00	.00	1.00
									1.00
.00	.00	.00	.00	.00	.00	.00	.00	.00	1.00
1.00	.00	.00	.00	.00	.00	.00	.00	.00	1.00
									2.00
									1.00
									1.00
.00	.00	.00	.00	.00	.00	.00	.00	.00	3.00
									4.00
									1.00
.00	.00	.00	.00	.00	.00	.00	.00	.00	1.00
1.00	.00	.00	.00	.00	.00	.00	.00	.00	2.00
									1.00
.00	.00	.00	.00	.00	.00	.00	.00	.00	1.00
.00	.00	.00	.00	.00	.00	.00	.00	.00	3.00
1.00	.00	.00	.00	.00	.00	.00	.00	.00	1.00
1.00	.00	.00	.00	.00	.00	.00	.00	.00	1.00
									3.00
.00	.00	.00	.00	.00	.00	.00	.00	.00	1.00
1.00	.00	.00	.00	.00	.00	.00	.00	.00	1.00
									3.00
.00	.00	.00	.00	.00	.00	.00	.00	.00	1.00
									1.00
									2.00
1.00	.00	.00	.00	.00	.00	.00	.00	.00	1.00
.00	.00	.00	.00	.00	.00	.00	.00	.00	1.00
									4.00
1.00	.00	.00	.00	.00	.00	.00	.00	.00	1.00
.00	.00	.00	.00	.00	.00	.00	.00	.00	1.00
.00	.00	.00	.00	.00	.00	.00	.00	.00	1.00
									1.00
									1.00
.00	.00	.00	.00	.00	.00	.00	.00	.00	1.00
.00	.00	.00	.00	.00	.00	.00	.00	.00	1.00
									3.00
									1.00
.00	.00	.00	.00	.00	.00	.00	.00	.00	1.00
									1.00
									4.00
									1.00
									3.00
.00	.00	.00	.00	.00	.00	.00	.00	.00	1.00
									1.00
									1.00

									1.00
.00	.00	.00	.00	.00	.00	.00	.00	.00	1.00
									1.00
.00	.00	.00	.00	.00	.00	.00	.00	.00	1.00
									1.00
.00	.00	.00	.00	.00	.00	.00	.00	.00	1.00
.00	.00	.00	.00	.00	.00	.00	.00	.00	4.00
.00	.00	.00	.00	.00	.00	.00	.00	.00	1.00
									1.00
.00	.00	.00	.00	.00	.00	.00	.00	.00	1.00
									2.00
1.00	.00	.00	.00	.00	.00	.00	.00	.00	1.00
.00	.00	.00	.00	.00	.00	.00	.00	.00	1.00
									5.00
									3.00
.00	.00	.00	.00	.00	.00	.00	.00	.00	1.00
									1.00
.00	.00	.00	.00	.00	.00	.00	.00	.00	1.00
									6.00
									1.00
.00	.00	.00	.00	.00	.00	.00	.00	.00	1.00
.00	.00	.00	.00	.00	.00	1.00	.00	.00	3.00
									1.00
.00	.00	.00	.00	.00	.00	.00	.00	.00	1.00
									1.00
.00	.00	.00	.00	.00	.00	.00	.00	.00	1.00
									4.00
									1.00
1.00	.00	.00	.00	.00	.00	.00	.00	.00	4.00
									4.00
.00	.00	.00	.00	.00	.00	.00	.00	.00	2.00
.00	.00	.00	.00	.00	.00	.00	.00	.00	2.00
									2.00
									1.00
									5.00
.00	.00	.00	.00	.00	.00	.00	.00	.00	3.00
									3.00
1.00	.00	.00	.00	.00	.00	.00	.00	.00	1.00
									1.00
									1.00
									3.00
									1.00
									2.00
.00	.00	.00	.00	.00	.00	.00	.00	.00	1.00
									1.00
.00	.00	.00	.00	.00	.00	.00	.00	.00	2.00
									5.00
1.00	.00	.00	.00	.00	.00	.00	.00	.00	3.00
									1.00
.00	.00	.00	.00	.00	.00	.00	.00	.00	1.00
									1.00
									1.00
									1.00
									1.00
									1.00
.00	.00	.00	.00	.00	.00	.00	.00	.00	1.00

.00	.00	.00	.00	.00	.00	.00	.00	.00	1.00
									2.00
									1.00
.00	.00	.00	.00	.00	.00	.00	.00	.00	3.00
									1.00
									1.00
									4.00
									4.00
									1.00
									2.00
									3.00
.00	.00	.00	.00	.00	.00	.00	.00	.00	1.00
1.00	.00	.00	.00	.00	.00	.00	.00	.00	1.00
.00	.00	.00	.00	.00	.00	.00	.00	.00	1.00
									5.00
.00	.00	.00	.00	.00	.00	.00	.00	.00	1.00
.00	.00	.00	.00	.00	.00	.00	.00	.00	1.00
									1.00
									4.00
									1.00
									1.00
.00	.00	.00	.00	.00	.00	.00	.00	.00	5.00
.00	.00	.00	.00	.00	.00	.00	.00	.00	1.00
.00	.00	.00	.00	.00	.00	.00	.00	.00	3.00
									3.00
									1.00
									3.00
.00	.00	.00	.00	.00	.00	.00	.00	.00	1.00
									3.00
.00	.00	.00	.00	.00	.00	.00	.00	.00	1.00
									1.00
.00	.00	.00	.00	.00	.00	.00	.00	.00	1.00
.00	.00	.00	.00	.00	.00	.00	.00	.00	1.00
.00	.00	.00	.00	.00	.00	.00	.00	.00	1.00
									1.00
									3.00
.00	.00	.00	.00	.00	.00	.00	.00	.00	1.00
									6.00
.00	.00	.00	.00	.00	.00	.00	.00	.00	1.00
									4.00
.00	.00	.00	.00	.00	.00	1.00	.00	.00	1.00
.00	.00	.00	.00	.00	.00	.00	1.00	.00	1.00
.00	.00	.00	.00	.00	.00	.00	.00	.00	1.00
.00	.00	.00	.00	.00	.00	.00	.00	.00	1.00
.00	.00	.00	.00	.00	.00	.00	.00	.00	1.00
.00	.00	.00	.00	.00	.00	.00	.00	.00	1.00
.00	.00	.00	.00	.00	.00	.00	.00	.00	1.00
.00	.00	.00	.00	.00	.00	.00	.00	.00	1.00
.00	.00	.00	.00	.00	.00	.00	.00	.00	1.00
									6.00
									3.00
.00	.00	.00	.00	.00	.00	.00	.00	.00	5.00
.00	.00	.00	.00	.00	.00	.00	.00	.00	1.00
									3.00
.00	.00	.00	.00	.00	.00	.00	.00	.00	1.00
									3.00

.00	.00	.00	.00	.00	.00	.00	.00	.00	1.00
.00	.00	.00	.00	.00	.00	.00	.00	.00	1.00
									1.00
.00	.00	.00	.00	.00	.00	.00	.00	.00	1.00
.00	.00	.00	.00	.00	.00	.00	.00	.00	1.00
.00	.00	.00	.00	.00	.00	.00	.00	.00	1.00
.00	.00	.00	.00	.00	.00	.00	.00	.00	1.00
.00	.00	.00	.00	.00	.00	.00	.00	.00	1.00
.00	.00	.00	.00	.00	.00	.00	.00	.00	1.00
.00	.00	.00	.00	.00	.00	.00	.00	.00	1.00
.00	.00	.00	.00	.00	.00	.00	.00	.00	1.00
.00	.00	.00	.00	.00	.00	.00	.00	.00	1.00
.00	.00	.00	.00	.00	.00	.00	.00	.00	1.00
.00	.00	.00	.00	.00	.00	.00	.00	.00	1.00
									1.00
.00	.00	.00	.00	.00	.00	.00	.00	.00	1.00
.00	.00	.00	.00	.00	.00	.00	.00	.00	1.00
									1.00
									6.00
.00	.00	.00	.00	.00	.00	.00	.00	.00	1.00
.00	.00	.00	.00	.00	.00	.00	.00	.00	1.00
.00	.00	.00	.00	.00	.00	.00	.00	.00	1.00
									3.00
.00	.00	.00	.00	.00	.00	.00	.00	.00	1.00
.00	.00	.00	.00	.00	.00	.00	.00	.00	3.00
.00	.00	.00	.00	.00	.00	.00	1.00	.00	1.00
.00	.00	.00	.00	.00	.00	.00	.00	.00	1.00
.00	.00	.00	.00	.00	.00	.00	.00	.00	3.00
.00	.00	.00	.00	.00	.00	.00	.00	1.00	1.00
.00	.00	.00	.00	.00	.00	.00	.00	.00	1.00
.00	.00	.00	.00	.00	.00	.00	.00	.00	3.00
.00	.00	.00	.00	.00	.00	.00	.00	.00	1.00
									1.00
.00	.00	.00	.00	.00	.00	.00	.00	.00	1.00
.00	.00	.00	.00	.00	.00	.00	.00	.00	4.00
.00	.00	.00	.00	.00	.00	.00	.00	.00	1.00
.00	.00	.00	.00	.00	.00	.00	.00	.00	1.00
									1.00
1.00	.00	.00	.00	.00	.00	.00	.00	.00	3.00
1.00	.00	.00	.00	.00	.00	.00	.00	.00	1.00
.00	.00	.00	.00	.00	.00	.00	.00	.00	1.00
									1.00
.00	.00	.00	.00	.00	.00	.00	.00	.00	1.00
1.00	.00	.00	.00	.00	.00	.00	.00	.00	1.00
									3.00
.00	.00	.00	.00	.00	.00	.00	.00	.00	1.00
									1.00
.00	.00	.00	.00	.00	.00	.00	.00	.00	2.00
.00	.00	.00	.00	.00	.00	.00	.00	.00	1.00
.00	.00	.00	.00	.00	.00	.00	.00	.00	1.00
.00	.00	.00	.00	.00	.00	.00	.00	.00	1.00
.00	.00	.00	.00	.00	.00	.00	.00	.00	1.00
1.00	.00	.00	.00	.00	.00	.00	.00	.00	1.00
.00	.00	.00	.00	.00	.00	.00	.00	.00	1.00
.00	.00	.00	.00	.00	.00	.00	.00	.00	1.00
.00	.00	.00	.00	.00	.00	.00	.00	.00	1.00

									1.00
									2.00
.00	.00	.00	.00	.00	.00	.00	.00	.00	1.00
1.00	.00	.00	.00	.00	.00	.00	.00	.00	1.00
.00	.00	.00	.00	.00	.00	.00	.00	.00	1.00
.00	.00	.00	.00	.00	.00	.00	.00	.00	3.00
.00	.00	.00	.00	.00	.00	.00	.00	.00	1.00
1.00	.00	.00	.00	.00	.00	.00	.00	.00	1.00
1.00	.00	.00	.00	.00	.00	.00	.00	.00	1.00
									1.00
.00	.00	.00	.00	.00	.00	.00	.00	.00	1.00
.00	.00	.00	.00	.00	.00	.00	.00	.00	1.00
									1.00
									1.00
									1.00
									1.00
1.00	.00	.00	.00	.00	.00	.00	.00	.00	1.00
									1.00
									2.00
									3.00
1.00	.00	.00	.00	.00	.00	.00	.00	.00	3.00
									5.00
.00	.00	.00	.00	.00	.00	.00	.00	.00	3.00
									3.00
									3.00
.00	.00	.00	.00	.00	1.00	.00	.00	.00	3.00
									2.00
									4.00
.00	.00	.00	.00	.00	.00	1.00	.00	.00	4.00
.00	.00	.00	.00	.00	.00	.00	.00	.00	2.00
									3.00
.00	.00	.00	.00	.00	.00	.00	.00	.00	3.00
.00	.00	.00	.00	.00	.00	.00	.00	.00	2.00
.00	.00	.00	.00	.00	.00	1.00	.00	.00	4.00
.00	.00	.00	.00	.00	.00	.00	.00	.00	3.00
									3.00
									4.00
									3.00
									2.00
									3.00
									1.00
									1.00
									2.00
									1.00
									3.00
									3.00
									3.00
									3.00
									2.00
									2.00
									3.00
									2.00
									2.00
									2.00
									1.00
									3.00

1. 00
2. 00
3. 00
3. 00
1. 00
2. 00
2. 00
3. 00
3. 00
1. 00
3. 00
4. 00
1. 00
1. 00
1. 00
4. 00
3. 00
4. 00
2. 00
3. 00
3. 00
3. 00
1. 00
1. 00
2. 00
2. 00
1. 00
1. 00
2. 00
3. 00
4. 00
1. 00
2. 00
1. 00
3. 00
3. 00
3. 00
1. 00
4. 00
3. 00
3. 00
3. 00
3. 00
3. 00
2. 00
2. 00
6. 00
3. 00
3. 00
1. 00
1. 00
3. 00
3. 00
2. 00
1. 00
1. 00

2. 00
2. 00
1. 00
3. 00
1. 00
2. 00
1. 00
1. 00
1. 00
1. 00
1. 00
3. 00
3. 00
3. 00
2. 00
1. 00
3. 00
1. 00
3. 00
1. 00
1. 00
1. 00
1. 00
1. 00
1. 00
2. 00
4. 00
3. 00
6. 00
1. 00
3. 00
1. 00
1. 00
2. 00
1. 00
1. 00
1. 00
1. 00
1. 00
2. 00
4. 00
1. 00
1. 00
1. 00
1. 00
3. 00
4. 00
2. 00
1. 00
3. 00
2. 00
2. 00
1. 00
1. 00
3. 00
2. 00

2. 00
2. 00
5. 00
2. 00
2. 00
3. 00
3. 00
3. 00
1. 00
2. 00
4. 00
2. 00
3. 00
1. 00
3. 00
3. 00
4. 00
1. 00
4. 00
1. 00
3. 00
3. 00
1. 00
5. 00
3. 00
3. 00
1. 00
2. 00
3. 00
5. 00
1. 00
3. 00
3. 00
3. 00
1. 00
4. 00
3. 00
3. 00
1. 00
1. 00
2. 00
3. 00
3. 00
1. 00
3. 00
4. 00
3. 00
1. 00
1. 00
3. 00
3. 00
3. 00
1. 00
1. 00
4. 00
3. 00

3. 00
4. 00
2. 00
1. 00
3. 00
3. 00
1. 00
3. 00
3. 00
6. 00
3. 00
1. 00
1. 00
3. 00
3. 00
3. 00
1. 00
1. 00
3. 00
4. 00
1. 00
5. 00
2. 00
4. 00
3. 00
3. 00
3. 00
5. 00
5. 00
3. 00
1. 00
1. 00
3. 00
2. 00
4. 00
1. 00
2. 00
5. 00
3. 00
1. 00
4. 00
3. 00
4. 00
1. 00
3. 00
2. 00
1. 00
2. 00
2. 00
4. 00
3. 00
3. 00
2. 00
1. 00
3. 00
3. 00

3. 00
3. 00
2. 00
1. 00
1. 00
5. 00
3. 00
5. 00
3. 00
3. 00
4. 00
6. 00
2. 00
5. 00
2. 00
1. 00
3. 00
2. 00
2. 00
1. 00
3. 00
1. 00
2. 00
1. 00
3. 00
3. 00
2. 00
3. 00
4. 00
